# Recent Progress in the Development of Advanced Functionalized Electrodes for Oxygen Evolution Reaction: An Overview

**DOI:** 10.3390/ma14164420

**Published:** 2021-08-06

**Authors:** Tse-Wei Chen, Palraj Kalimuthu, Ganesan Anushya, Shen-Ming Chen, Vinitha Mariyappan, Rasu Ramachandran

**Affiliations:** 1 Department of Materials, Imperial College London, London SW7 2AZ, UK; t.chen19@imperial.ac.uk; 2 School of Chemistry and Molecular Biosciences, University of Queensland, Brisbane 4072, Australia; p.kalimuthu@uq.edu.au; 3 Department of Physics, S.A.V. Sahaya Thai Arts and Science (Women) College, Sahayam Nagar, Kumarapuram Road, Vadakkankulam, Tirunelveli 627116, India; anushya@savsahayathaicollege.com; 4 Electroanalysis and Bioelectrochemistry Lab, Department of Chemical Engineering and Biotechnology, National Taipei University of Technology, No.1, Section 3, Chung-Hsiao East Road, Taipei 106, Taiwan; vinithavicky80@gmail.com; 5 Department of Chemistry, The Madura College, Vidya Nagar, Madurai 625011, India

**Keywords:** advanced electrocatalysts, fabrication technique, nanocomposite, oxygen evolution reaction, cyclic stability

## Abstract

Presently, the global energy demand for increasing clean and green energy consumption lies in the development of low-cost, sustainable, economically viable and eco-friendly natured electrochemical conversion process, which is a significant advancement in different morphological types of advanced electrocatalysts to promote their electrocatalytic properties. Herein, we overviewed the recent advancements in oxygen evolution reactions (OERs), including easy electrode fabrication and significant action in water-splitting devices. To date, various synthetic approaches and modern characterization techniques have effectively been anticipated for upgraded OER activity. Moreover, the discussed electrode catalysts have emerged as the most hopeful constituents and received massive appreciation in OER with low overpotential and long-term cyclic stability. This review article broadly confers the recent progress research in OER, the general mechanistic approaches, challenges to enhance the catalytic performances and future directions for the scientific community.

## 1. Introduction

There have been significant developments of clean and green energy and more efficient renewable sources, which include electrochemical conversions and storage methods in the fields of solar cells [1], fuel cells [2] and batteries [3], etc. To reach the next level energy conversion process, there needs to be a cost-effective manner for sustainable energy, which can fulfil the global energy demands [4]. High performance-based novel types of electrode catalysts have been developed for oxygen evolution reactions (OERs), specifically in oxides [5], hydroxides [6], sulfides [7] and phosphides [8]. These types of electrode catalysts have been specifically designed to achieve more active electrode surfaces to boost their electrocatalytic activities during the electrochemical process. The general mechanism of major important electrochemical energy devices for the hydrogen and oxygen evolution reactions are the key parameters that occur at both the anode and cathode electrode materials for hydrogen production through the water-splitting process, which is discussed in more detail in Section 5 [9].

**Scheme 1:**
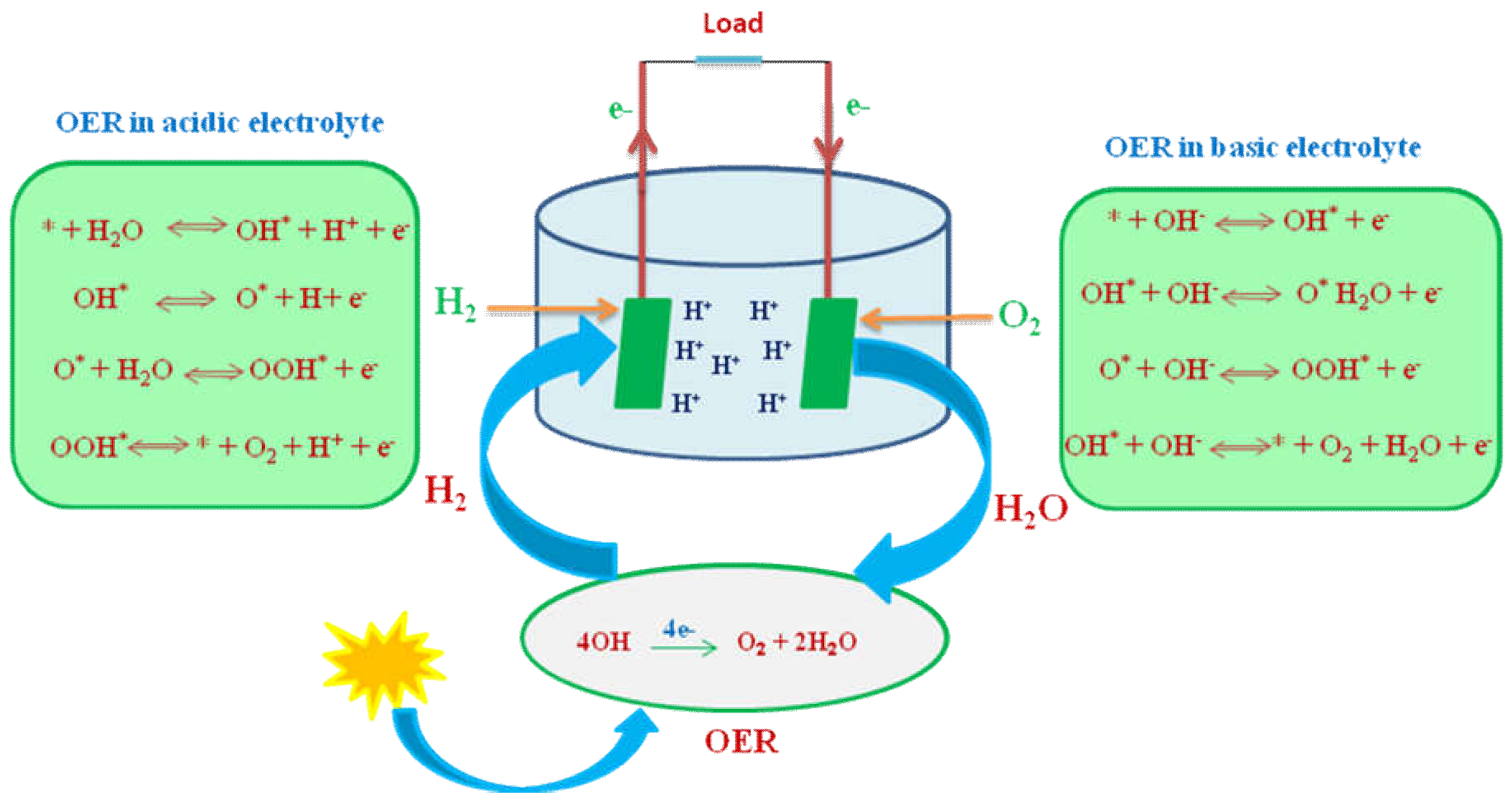
Schematic representation of oxygen evolution reaction (OER) by water hydrolysis (the activated molecules is denoted by the symbol * in the above scheme).

Yuan Li and co-workers [10] electrochemically fabricated the microporous bi-continuous structure of phosphorous-doped (P-(PbO_2_-MnO_2_)) composite electrodes for the study of OER activities, and the possible oxygen evolution mechanism was also highlighted. Ahigh surfacearea-based iridium (Ir) scaffold with activated carbon composite electrocatalysts was prepared by pyrolysis method, with the synergetic effect of this composite as the electrode for oxygen reduction reaction (ORR) in seawater electrolysis [11]. At present, novel 2D-structured NiO/CeO_2_ bi-functional electrocatalysts have been studied in OER activities, because of the large contact area they provide, thereby improving the adsorption and splitting of water molecules [12]. Yang and co-workers demonstrated that novel and highly stable tri-metallic NiFeCr electrocatalysts enhance the intrinsic OER catalytic activity and long-term cyclic durability during OER studies [13]. The bi-functional natures of hollow structured Ni@NiCo_2_O_4_ electrocatalysts have received considerable attention for OER studies. However, the constructed novel electrocatalyst can exhibit a high surface area, faster electron transport and anachieved current density value of 10 mA cm^−2^ [14]. Li et al. [15] studied a nitrogen-doped cobalt oxide (N-Co_3_O_4_) electrode catalyst as a potential candidate for OER. Here, the doping mechanism of nitrogen with metal oxides can significantly improve the OH^−^ adsorption capability, increasing electronic conductivity and excellent OER performances with good cyclic stability. Broicher et al. [16] showed an overpotential value of 385 mV with a Tafel slope ranges from 43 to 199 mV dec^−1^ at the recorded current density value of 10 mA cm^−2^ for a spinel structure of a mesoporous nickel cobalt oxide (NiCo_2_O_4_) catalyst. The highly desirable, active and robust vertically aligned cobalt-molybdenum-supported gold (AuNPs/CoMoN*_x_*) nanocomposite was explored with a large accessible surface area and high intrinsic activity. The synthesized nanocomposite facilitates fast electron transfer process with remarkable OER catalytic activity under alkaline conditions [17]. A fast and facile heterogeneous-based tannin-NiFe(TANF) complex film was fabricated, which can be modified with carbon paper (CP). After the fabrication process, TA-metal complex film composites were considered as the new-type OER active electrocatalysts [18]. Interestingly, a nitrogen-enriched graphene quantum dots (C-GQDs) catalyst was developed with an efficient electrocatalytic activity, and the charge distribution of pyridine and pyridine N-oxide is induced through the π–π delocalization process, which could encourage their OER activity [19]. Surendran et al. [20] hydrothermally synthesized the spherically nanostructured carbon-enriched cobalt phosphide multi-functional electrode for water electrolyzer studies, and reported the current density of 10 mA cm^−2^ @ 1.63 V. Qian et al. [21] prepared a cost-effective, larger specific surface area with high electrically conductive natured MWCNTs for the making of a thermo-electrochemical (TEC) catalyst by the electrochemical deposition (EPD) method. The prepared MWCNT TEC catalyst could display a high power density (45.2 A cm^−2^) with long-term cyclic stability. The high synergetic-based MWCNT bucky paper and MWCNT forest electrode materials were used for the harvesting of high thermo-electrochemical cells from waste thermal energy resources. Moreover, the MWCNT forest temperament electrode significantly enhanced the thermo cell efficiency by 30% which is more than that of the MWCNT bucky paper electrode [22]. The OER is a promising energy storage device and for the production of fuel H_2_ based on the water splitting process (Scheme 1) [9]. Table S1 presents synthesis routes, electrolytes and some of the important electrochemical parameters related to the advanced electrocatalysts for OER.

In this regard, the current advancement research in OER studies, using functionalized, highly electroactive catalysts, are summarized, which include the design, detailed mechanism, characterization, evaluation and various techniques used for assembling advanced electrocatalysts and also sensibly increasing their electrocatalytic properties, whichwill help the scientific community. Based on this fruitful discussion, we briefly present the current challenges and the significant achievements in the OER catalyst. The futuristic scopes of research strategies for determining electrocatalytic activity with the stability of advanced catalysts are highlighted.

## 2. Electrode Surface Area

**Figure 1:**
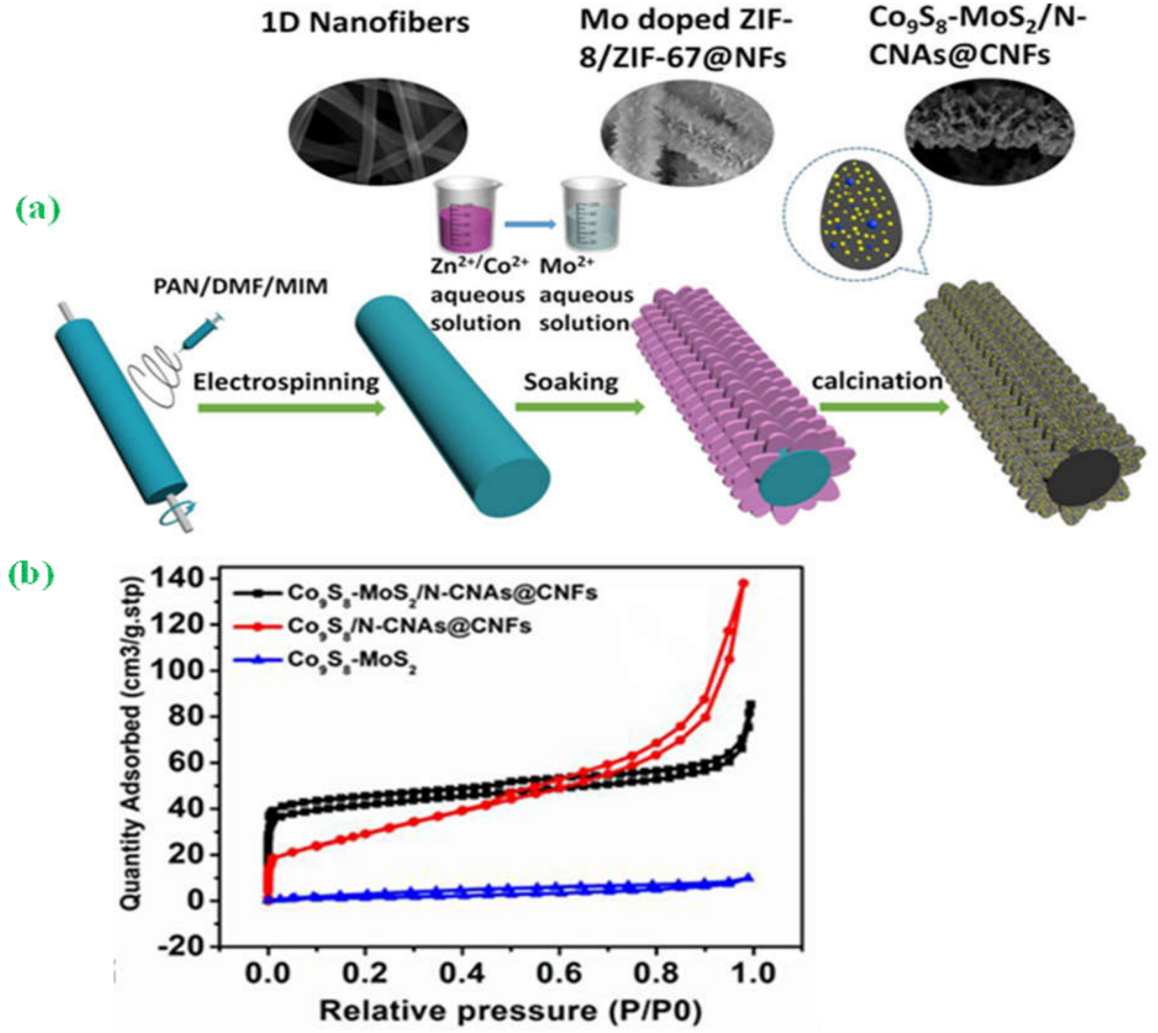
(**a**) Schematic illustration of the synthesis scheme of Co_9_S_8_-MoS_2_/NCNAs@CNFs and (**b**) N_2_ adsorption/desorption isotherms of samples. Copyright 2020 by the American Chemical Society [31].

**Figure 2:**
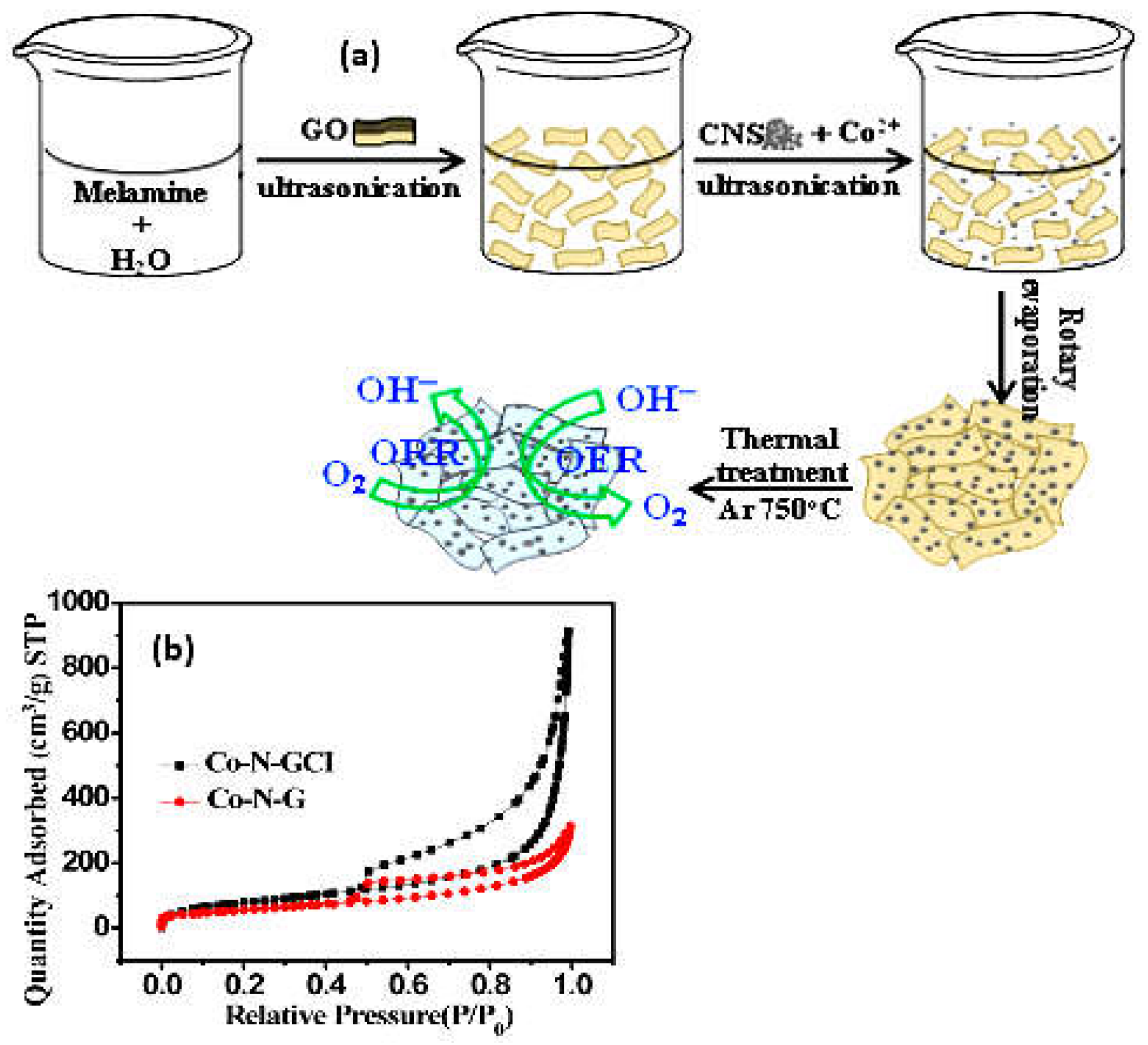
(**a**) Schematic illustration of the fabrication process for the Co-N-G catalyst along with effective catalytic performance towards OER and (**b**) nitrogenadsorption/desorption isotherms. Copyright 2016 by the American Chemical Society [32].

Electrode surface area is an important parameter to expose more electroactive sites and boost their electrocatalytic properties. N_2_adsorption/desorption studies, the pore size distribution and thenumber of available electroactive sites have been estimated by the Brunauer–Emmett–Teller (BET) and Barrett–Joyner–Halenda methods [23,24]. The porous structure of MoS_2_ has been synthesized by the hydrothermal method. Due to the largespecific surface area (17.5 m^2^ g^−^^1^), the catalyst represents a promising candidate for OER activity with enhanced cyclic stability [25]. Sharma et al. [26] used Co_3_O_4_ with fascinating morphologies of hierarchical nanosheets to have a better particle size and large surface area (37 m^2^ g^−^^1^),whichresulted in next-generation supercapacitor (~230 F g^−^^1^) energy storage device applications. The bi-functional natured CoP/HNCNP@CoP electrode matrix has been well established under certain conditions. The porous structured matrix has been analyzed by the N_2_ adsorption/desorption technique, and the estimated surface area value (156.04 m^2^ g^−^^1^) has been confirmed by the BET method and it could provide benefits for the energy conversion process [27]. The Ru-doped α-MnO_2_-based benchmark electrocatalysts were shown to be an excellent catalyst for OER, with a larger BET surface area (87.4 m^2^ g^−^^1^). This is clearly indicated by the size and shape of the particles. Furthermore, the most attractive bi-functional Ru-doped α-MnO_2_ catalyst could also be used for water splitting reactions [28] The 3D-conductive flake structure of iron-based nitrogen and nanocarbon-derived (Fe-NSDC) electrocatalysts shows a large surface area (1533.7 m^2^ g^−^^1^) with high microporous structure volume (0.39 cm^3^ g^−^^1^), and aresulting current density value of 100 mA cm^−^^2^ for a Zn-air battery [29]. Wahab et al. [30] developed a low-cost, metal-free and high surface area (406 m^2^ g^−^^1^) mesoporous-based graphitic carbon nitrite (gMesoCN) catalyst that showed good electrochemical performance for OER in alkaline conditions. Very recently, Zhang et al. [31] designed and synthesized a novel Co_9_S_8_and MoS_2_ nanocrystal-modified 3D nitrogen-doped carbon nanoflakes (Co_9_S_8_-MoS_2_/N-CNAs@CNFs) catalyst (Figure 1), which shows a high electrode surface area (178.46 m^2^ g^−^^1^); thereby it has more active sites and can act with good electrocatalytic activity towards OER. The state-of-the-art cobalt-supported nitrogen co-doped 3D graphene (Co-N-G) nanospheres have drawn special attention due to the large BET surface area (365.2 m^2^ g^−^^1^) determined from the N_2_ adsorption/desorption method and having an effective electrocatalyst performance in OER (Figure 2a,b) [32]. A class of ordered mesoporous-structured carbon-supported Co_3_O_4_ has been successfully synthesized by a soft-templating method. Herein, poly (ethylene oxide)-b-polystyrene can be used as a synthetic agent. In addition, the Co_3_O_4_ catalyst can act with superior electrocatalytic properties due to havingversatile properties like controlled morphologies, a larger BET electrode surface area (407 m^2^ g^−^^1^), along with higher OER catalytic activity [33].

## 3. Advanced Electrode Materials for OER

The major classes of electrode materials fabricated and successfully used for OER applications in the literature are related to metal oxides, polymer composites, porous carbon, conducting polymers, metal and carbon-based nanoparticles, graphene and carbon nanotubes [34]. In this section, we focused on the recent development of new electrode materials used for the generation of O_2_through the electrochemical oxidation of water in energy applications.

### 3.1. Nanocarbon-Based Electrodes

NiCo_2_O_4_ (NC)-carbon dots (CDs) nano needle arrays have been fabricated on nickel (Ni) foam and directly used as an OER catalyst without any electrode binder [35]. Poly(vinyl pyrrolidone) were exposed to heat for 5 h at 180 °C to obtain CDs, and then different amount (5, 25 and 40 mg) of CDs were mixed with another couple of salts to generate the catalysts, and they were denoted as NCCD5, NCCD25 and NCCD40, respectively. The developed materials were applied for OER activity in alkaline media (1 M KOH) and it was found that NC, NCCD5, NCCD25 and NCCD40 exhibited the onset potential of 510, 460, 390 and 470 mV vs. RHE, respectively. It is clear that the catalytic response significantly improved upon the incorporation of CDs into the NC. However, the onset potential increased upon loading a higher concentration of CDs (40 mg) due to the excessive number of CDs, which induced aggregation. Further, the Tafel slope of the catalysts was examined to be 140, 95 and 165 mV dec^−1^ for NCCD5, NCCD25 and NCCD40, respectively, and this indicates that a more effective electron transfer occurs in NCCD25 than the other two materials. For practical applicability, the electrochemical durability of the materials was evaluated by continuous run the potential window between 1.1 to 1.9 V vs. RHE for 1000 cycles. NCCD25 displayed stable OER performances of about >98.5%, whereas NC maintained ~95% retention of the initial potential. These results supported that the developed hierarchical NCCD nanohybrid provides a higher chargetransfer rate along with improved electrocatalytic performances and stability owing to the presence of a larger number of active sites.

**Figure 3:**
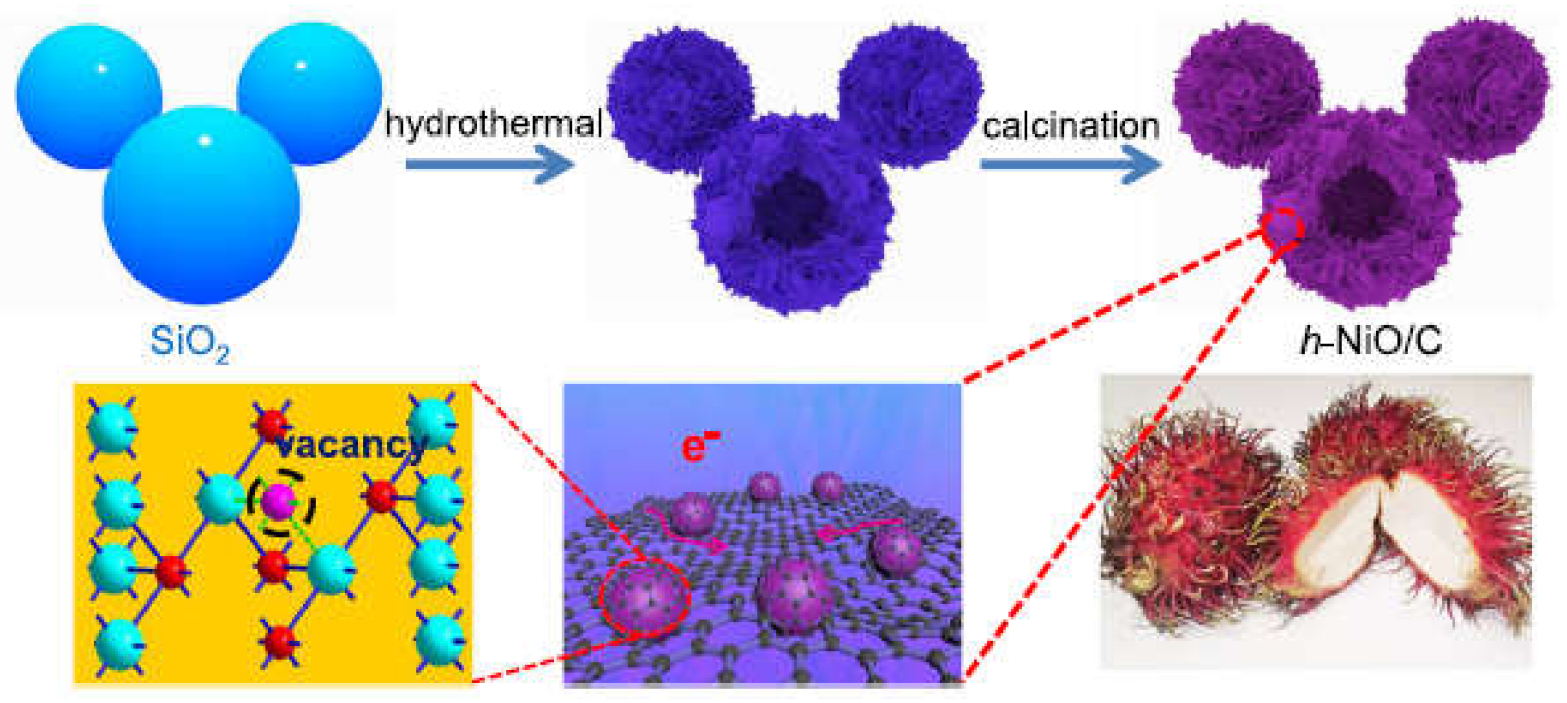
The fabrication process of *h*-NiO/C. The as-prepared *h*-NiO/C sample with abundant oxygen vacancies looks like a rambutan shell. Inset: a photo of rambutans. Copyright 2019 by Wenzhou University and John Eiley andSons Australia, Ltd. [36].

A bifunctional electrode material for OER was reported based on carbon nanosheet-decorated nickel oxide (NiO), and it appears as a rambutan-like hollow carbon sphere, as shown in Figure 3 [36]. A simple hydrothermal method was used to generate the hollow-structured precursor, and next, the precursor was calcinated under the anaerobic condition at 320 °C for 2 h. Finally, NiO nanoparticle-decorated hollow carbon nanosheets (*h*-NiO/C) with the size of 2 nm were obtained. The catalytic efficiency of *h*-NiO/Cwas evaluated in 1 M KOH and the performances were compared with its precursor *h*-NiO and the commercial catalyst RuO_2_. An overpotential of 260 mV was achieved at 10 mA cm^−2^ for the *h*-NiO/C electrode, which is significantly lower than the overpotential obtained for *h*-NiO (302 mV). Moreover, a smaller Tafel slope of 37.6 mV dec^−1^ was obtained for the *h*-NiO/C electrode compared to *h*-NiO (72.5 mV dec^−1^) and RuO_2_ (115.3 mV dec^−1^). This faster electrode kinetics imply that the synergistic effect between oxygen vacancies and the carbon framework could improve the OER performances.

### 3.2. Porous Carbon and Metal-Based Electrodes

Zhang and coworkers developed a 3D Ni foam/porous carbon/anodized Ni (NF/PC/AN) electrode for OER application. The fabrication of composite materials was carried out by a step-by-step process [37]. Firstly, to enhance the affinity of Ni foam, the oxide layer was removed by the acidified polyvinylpyrrolidone. Subsequently, Ni foam was soaked into the mixture of zinc nitrate and 2-methylimidazole (ZIF-8) and deposited on the polyhedral crystal membrane with a thickness of 4.5 µm. The resulting material underwenta calcinations process at 800 °C for 5 h to convert it into Ni foam/porous carbon. Finally, anodization was performed at a constant applied potential of 1 V vs. Ag/AgCl in 0.1 M KOH for 2 h to attain the 3D NF/PC/AN electrode material. The as-prepared material was tested to the OER activity. The 3D NF/PC/AN electrode showed a weak anodic and cathodic wave at 470 and 445 mV attributed to the Ni(OH)_2_/NiOOH redox reaction. Further, a pronounced catalytic wave emerged with an onset potential of 560 mV. The observed catalytic response was significantly higher than the Ni foam electrode. The authors mentioned that the porous carbon membrane acts as an interlayer between the active surface and conductive substrate for efficient electron transport to promote the OER activity.

As reported, the CoMoN*x* is thermodynamically unstable and easily oxidized to form electroactive CoMoO*x* at a higher potential. Thus, CoMoN*x* nanosheets are vertically aligned on NP-Au, and it becomes largely accessible for the catalytic reaction. The fabricated catalyst assessed the OER capability and the catalytic response compared with its precursor coated electrodes such as Au/CoMoO_4_ and Au/CoNx, and also the commercial catalyst Au/RuO_2_. To check the importance of nanoporous Au, the CoMoN*x* was coated on a glassy carbon electrode using a Nafion polymer binder.

**Figure 4:**
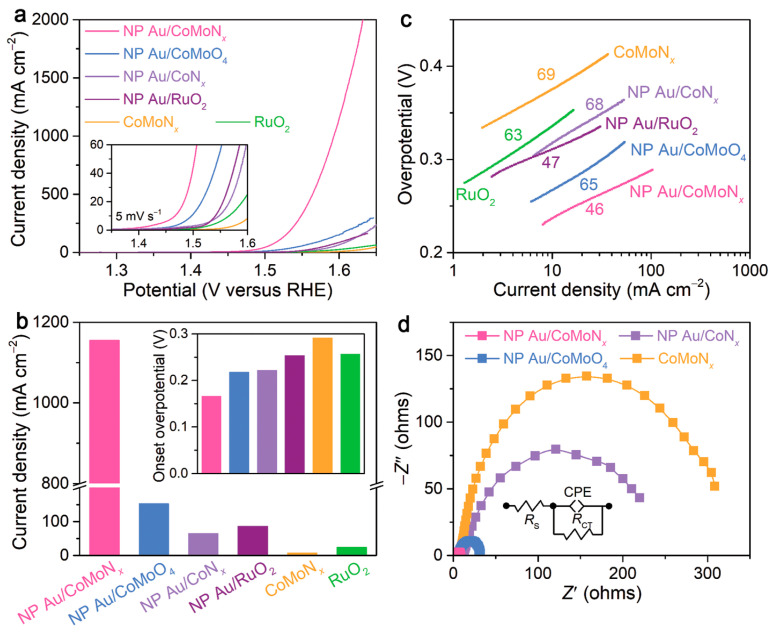
OER activity of bimetallic cobalt-molybdenum nitride nanosheets vertically aligned on NP-Au electrodes in alkaline media. (**a**) OER polarization curves of NP-Au/CoMoN*x*, Au/CoMoO_4_, Au/CoN*x* and Au/RuO_2_, as well as free CoMoN*x* nanosheets and RuO_2_ immobilized on a GC electrode at 5 mV s^−1^ in 1 M KOH. Inset: Expanded view of the lowoverpotential region in panel (**b**). Comparison of the current densities at the overpotential of 0.37 V for the electrodes shown in panel (a). Inset: The onset overpotentials of these different electrocatalysts for the OER. (**c**) Tafel plots comparing the Tafel slopes of different catalysts obtained from the polarization curves in panel (**a**). (**d**) EIS spectra of electrodes shown in panel (**a**). Copyright 2020 by John Wiley and Sons [38].

As shown in Figure 4a, NP-Au/CoMoN*x* exhibited a remarkable catalytic response compared to all other electrode materials. In addition, NP-Au/CoMoNx achieved a low Tafel slope of 46 mV dec^−1,^ which is marginally lower than the commercial Au/RuO_2_ (47 mV dec^−1^) (Figure 4b). These superior catalytic performances are associated with the fast electron transfer of highly conductive CoMoN*x* [38] The durability of the NP-Au/CoMoN*x* electrode was evaluated by measuring continuously for 50 h at the current densities of 10 and 50 mA cm^−2^, and it was found that the overpotential remained stable at 10 mA cm^−2^, whereas the overpotential slightly increased at 50 mA cm^−2^ as a result of the decreasing aqueous electrolyte concentration.

Very recently, Kumar et al., reported a coral-shaped NiCo_2_O_4_ nanostructure catalyst for bi-functional activities [39]. Interestingly, the coral-shaped nanostructure was developed by growing less stable and highly reactive (111) planes of NiCo_2_O_4_ single crystals (32 to 35 nm) at the (100) planes in the ⟨111⟩ direction to minimize the total interfacial free energy and thereby get attached to each other and form a chain-like nanostructure. The authors mentioned that the aging process plays a vital role in catalytic performances of the catalyst due to the increase of the particle and agglomeration size of the nanostructure.

Based on varying the aging time between 6 and 24 h, several samples were prepared and they were denoted as NCO6, NCO12, NCO18 and NCO24 for 6 h, 12 h, 18 h and 24 h of aging time, respectively. The developed electrode materials were tested for OER activity in 0.1 M KOH. Among the various aging times, the material prepared at 24 h (NCO24) exhibited a superior catalytic response due to its highly porous coral-shaped nature, and this catalytic activity was compared with the commercial catalyst IrO_2_. NCO24 displayed 30 mV onset potential and 290 mV overpotential at 10 mA/cm^2^ current density for OER compared to IrO_2,_ which offers 310 mV. Additionally, the Tafel slope of 102 mV/dec was estimated for NCO24, which is comparable with the value obtained for the IrO_2_ sample (97 mV/dec).

**Figure 5:**
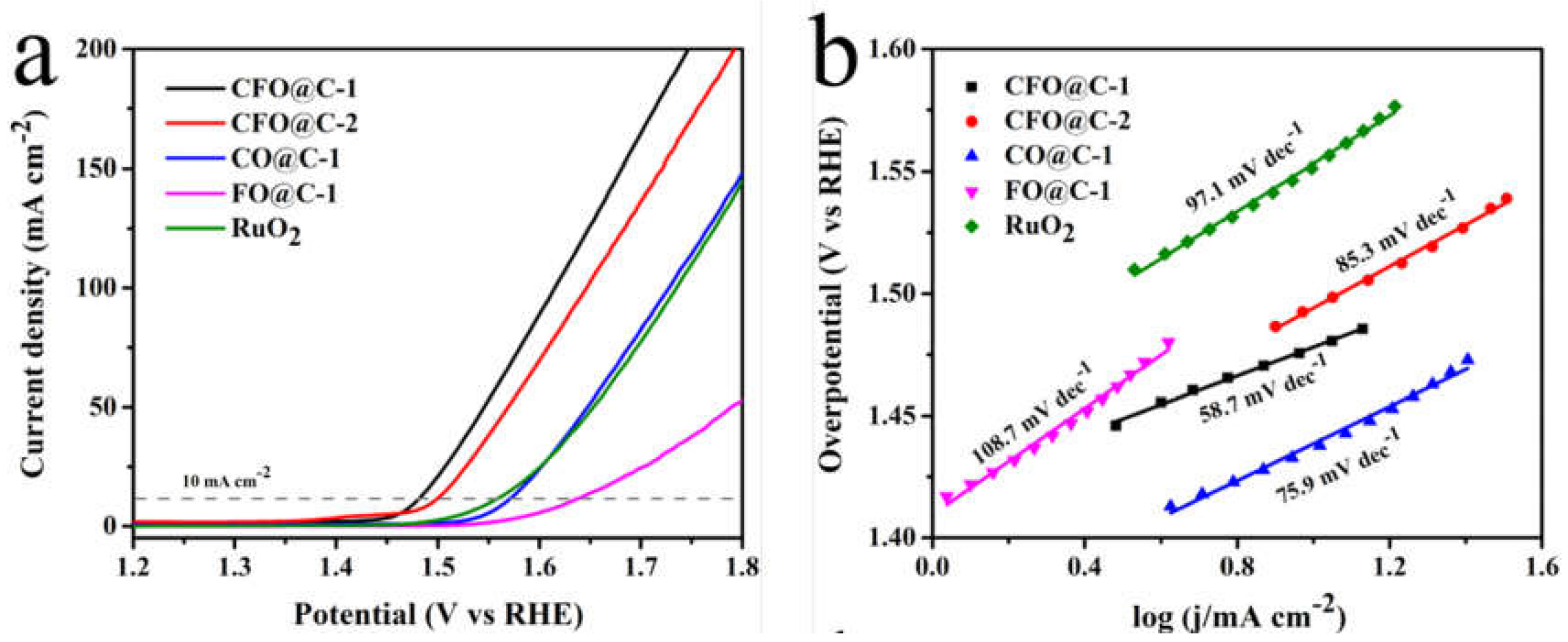
OER activity of mesoporous CoFe-based nanomaterials in 0.1 M KOH. (**a**) LSV curves and (**b**) Tafel slopes for CFO@C-1, CFO@C-2, CO@C-1, FO@C-1 and RuO_2_. Copyright 2020 by Elsevier [40].

The solvothermal process was employed to design the mesoporous CoFe-based nanomaterials for OER activity [40]. The OER performance was examined by the varying pore size of materials. Basically, two mesoporous materials with different pore sizes were developed by the incorporation of surfactants, namely hexadecyltrimethylammonium bromide (CTAB) and polyethylene glycol 4000 (PEG-4000), and the constructed materials were referred to as CFO@C-1 and CFO@C-2, respectively. It was revealed that CFO@C-1 has more pores than CFO@C-2. The average pore size was found to be 29.3 nm and 10.1 nm for CFO@C-1 and CFO@C-2, respectively. The catalytic performance of the electrode materials was investigated and compared with the monometallic counterparts (CO@C-1 and FO@C-1)and also to the commercial catalyst RuO_2_. As shown in Figure 5, CFO@C-1 exhibits better catalytic activity compared with CFO@C-2, CO@C-1, FO@C-1 and RuO_2_. CFO@C-1 showed the overpotential of 248 mV at 10 mA·cm^−2^ for OER compared to CFO@C-2 (265 mV), CO@C-1 (338 mV), FO@C-1 (400 mV) and RuO_2_ (324 mV). In addition, CFO@C-1 exhibited a lower Tafel slope of 58.7 mV·dec^−1^ compared to CFO@C-2 (85.3 mV dec^−1^), CO@C-1 (75.9 mV dec^−1^), FO@C-1 (108.7 mV dec^−1^) and RuO_2_ (97.1mV dec^−1^). These results clearly indicate that CFO@C-1 has much faster OER kinetics and more superior catalytic activity due to the presence of a higher number of pore sizes.

Chen and coworkers constructed a metal phosphide-based electrode material for OER activity [41]. A porous carbon membrane was prepared from Magnolia leaves and then used for the attachment of Fe-Co-P. The coating of Fe-Co-P on porous carbon was performed by electrochemical deposition, and this strong coupling promotes the stable electron and ion transport for OER. In addition, the pubescence fiber present in the Magnolia leaves can help to achieve efficient electron transport. OER activity was investigated for the prepared electrode material Fe-Co-P/C and also for its precursors, such as Fe-P/C and Co-P/C, in 1 M KOH. A pronounced OER catalytic response was achieved at Fe-Co-P/C, and it showed the overpotential of 151 mV at 10 mA cm^−2^. On the other hand, the overpotentials of 218 mV, 186 mV and 270 mV were achieved at Fe-P/C, Co-P/C and pristine porous carbon, respectively. Moreover, Fe-Co-P/C displayed the Tafel slope of 77.78 mV dec^−1^, which was significantly smaller than Fe-P/C (88.24 mV dec^−1^) and Co-P/C (104.22 mV dec^−1^). These results revealed the strong interaction between the porous carbon membrane and bimetallic phosphides, as well as the coupling effect between the Co and Fe elements, which remarkably increased the active sites.

## 4. Functionalized Electrode Materials for OER

The functionalization surface could modify the chemistry of the electrode material surface to provide specific properties related to the specified applications. Various functionalization methods employed in the literature as well as the primary outcomes of this process change the materials’ surface energy. This modification can help for better coupling with other targeted substances at the molecular level [42,43,44]. Nowadays, the functionalization process is an unavoidable step at the basis of the preparation of efficient electrode materials. This section focuses on the electrode materials designed for OER applications related to functionalization with the polymer nanocomposite and doping of metals and non-metals.

### 4.1. Nanocomposite-Based Electrodes

Manganese oxide-integrated carbon (MnO/C) nanorod arrays were synthesized from Mn-metal-organic frameworks (MOFs) nanoarrays [45]. The hydrothermal method was utilized to grow Mn-MOF-74 nanorod array precursors directly on Ni foam substrate by the reaction of 2,5-dihydroxyterephthalic acid and manganese chloride tetrahydrate. Then, the annealing proceeds to sublimate the organic linker convert as a MnO nanorod array without any carbon component, which is denoted as MnO/C.In the calcination process, the temperature varied from 300 °C to 900 °C to convert Mn-MOF-74 into MnO/C and, therefore, the obtained materials were referred to as MnO/C-300/350/450/550/650/750, respectively. The developed materials were successfully applied for OER application. Overall, the MnO/C-T materials displayed a better performance compared to the pure MnO electrode. Particularly, MnO/C-350 exhibited the superior OER response along with a low overpotential (368 mV) at the current density of 20 mA cm^−2^ compared to the other MnO/C-T electrodes. Additionally, the catalytic response of MnO/C-350 compared with the commercial IrO_2_ achieved the overpotential of 481 mV.

**Figure 6:**
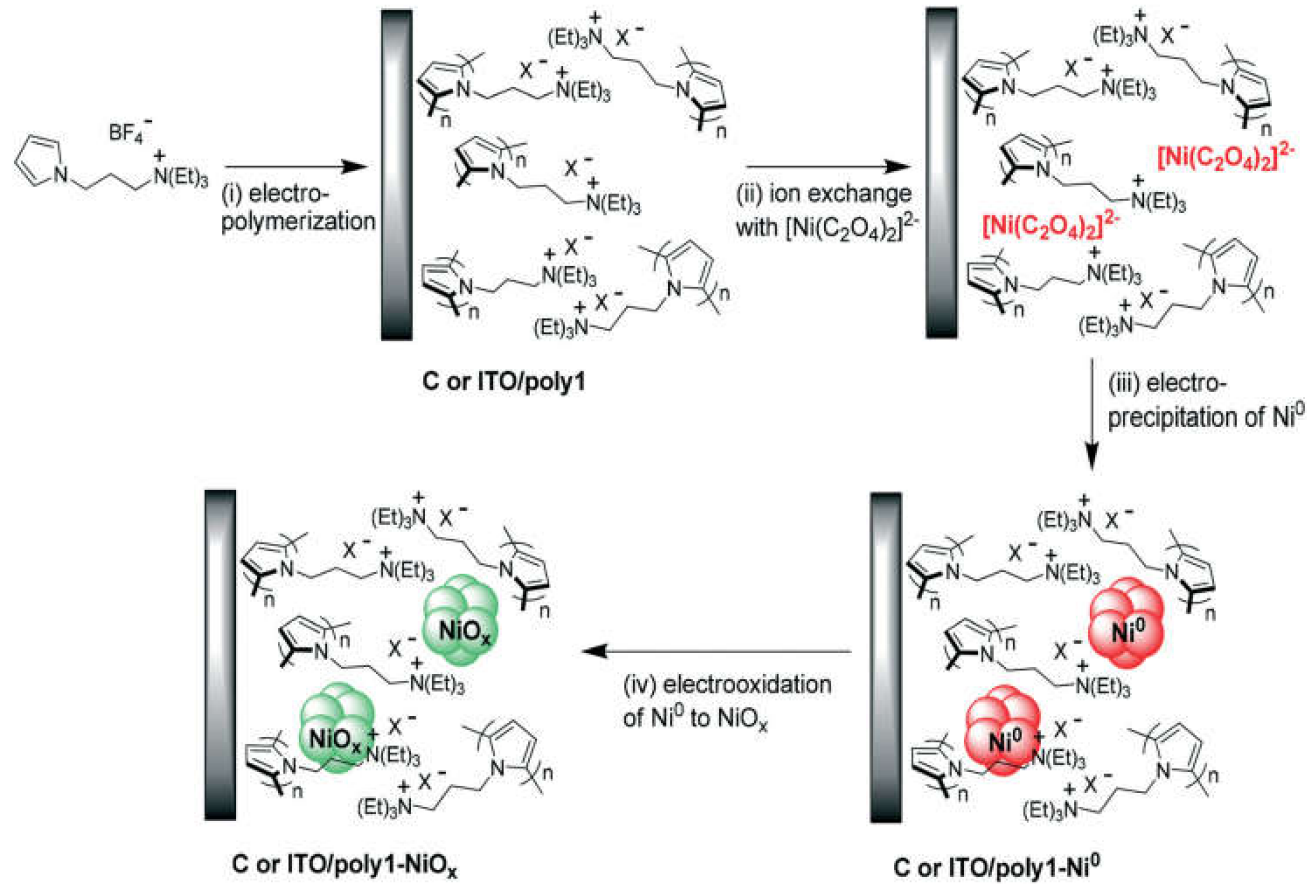
Electrosynthesis of poly(pyrrole-alkylammonium)/NiO*x* composite film-modified electrodes. (i) Formation of poly1 by oxidative electro-polymerization of monomer 1 (4 mM) at E_app_ = +0.95 V vs. Ag/AgNO_3_ in CH_3_CN, 0.1 M [Bu_4_N]ClO_4_; (ii) incorporation of [Ni(C_2_O_4_)_2_]^2−^ into poly1 upon soaking for 1 h in an aqueous solution (pH 6) of 1 mM NiSO_4_, 5 mM NaC_2_O_4_, 0.1 M Na_2_SO_4_ and 0.1 M H_3_BO_3_; (iii) electro-precipitation of Ni^0^ within poly1 at E_app_ = −1.4 V vs. Ag/AgCl in the previously mentioned aqueous solution of nickel oxalate; (iv) electrooxidation of Ni^0^ to NiO_x_ by repeated cyclic voltammetry scans between 0 and +1.2 V vs. Ag/AgCl in an aqueous solution (pH 9.2) of 0.1 M H_3_BO_3_ and 0.05 M NaOH. Copyright 2018 by Royal Society of Chemistry [46].

An electrochemical method was performed to fabricate the nanocomposite electrode materials for OER application insitu based on NiO nanoparticles entrapped in a polypyrrole matrix. The preparation protocol is given in Figure 6 [46]. Firstly, the cationic poly(pyrrole-ammonium) film (poly1) was electrochemically deposited with an applied potential of +0.95 vs. Ag/AgNO_3_on carbon or an ITO electrode, and then, using the ion-exchange method, a BF_4_^−^ counter ion was replaced with the anionic nickel oxalate complex, [Ni(C_2_O_4_)_2_]^2−^. Subsequently, the nickel oxalate was converted into Ni^0^ nanoparticles by electrochemical reduction at an applied potential of −1.4 V vs. Ag/AgCl.To compare the role of the poly1 film coating, Ni^0^ nanoparticles were deposited on the bare carbon electrode. The deposited film showed a well-defined redox response around 0.74 V, corresponding to the Ni^II^(OH)_2_/Ni^III^(O)(OH) redox couple [47].

The catalytic performance of C/poly1–NiO*x* and C/NiO*x* electrodes was examined for OER activity at pH 9.2, 13 and 14.Interestingly, the developed electrodes are catalytically active at pH 9.2, which is very important for use for natural water samples and also other applications like photoelectrochemical cells, where the molecular photosensitizer is often poorly stable in strongly basic solutions [48]. Further, the catalytic current drastically increased upon increasing the pH from 9.2 to 14. Additionally, the overpotential decreased from 0.61 to 0.35 V.

### 4.2. Non-Metal-Doped Electrodes

Nitrogen (N) doping in carbon materials could increase the positive charge of adjacent carbon atoms due to its stronger electronegativity, promoting the OER activity by the strong interaction between the OOH and N-doped site [49]. Zhang et al., employed the N-doping strategy to design the electrocatalyst N-doped spongy Ni (N-SN) for OER activity [50]. The as-prepared electrode material showed enhanced conductivity and an increased number of active sites, which offers remarkable OER performance. Further, N-SN achieved the low overpotential of 365 mV at 100 mA cm^−2^ and a small Tafel slope of 33 mV dec^−1^,outperforming the unmodified sponge Ni electrode that showed the overpotential of 549 mV with a Tafel slope of 40 mV dec^−1^.Further, N-doped carbon-functionalized CoSe_2_ nanowires (CoSe_2_@N–C NWs) were proposed for OER activity [51].The constructed CoSe_2_@N–C NWs attained a 6.61-fold higher OER catalytic response compared to the pristine CoSe_2_ NW electrode in 1.0 M KOH solution. This excellent catalytic response is attributed to the formation of an active CoO_x_H_y_/CoSe_2_@N–C NWs heterostructure. Based on the theoretical calculation, the authors pointed out that N-doping is beneficial for the optimization of free energy. In more details, the conversion of O* to OOH* at the pristine CoSe_2_ electrode resulted in the uphill potential of 1.80 eV with a high overpotential of 0.57 V, whereas the potential significantly decreased to 1.63 eV with a low overpotential of 0.40 V at N-doped CoSe_2_@N–C NWs.

**Figure 7:**
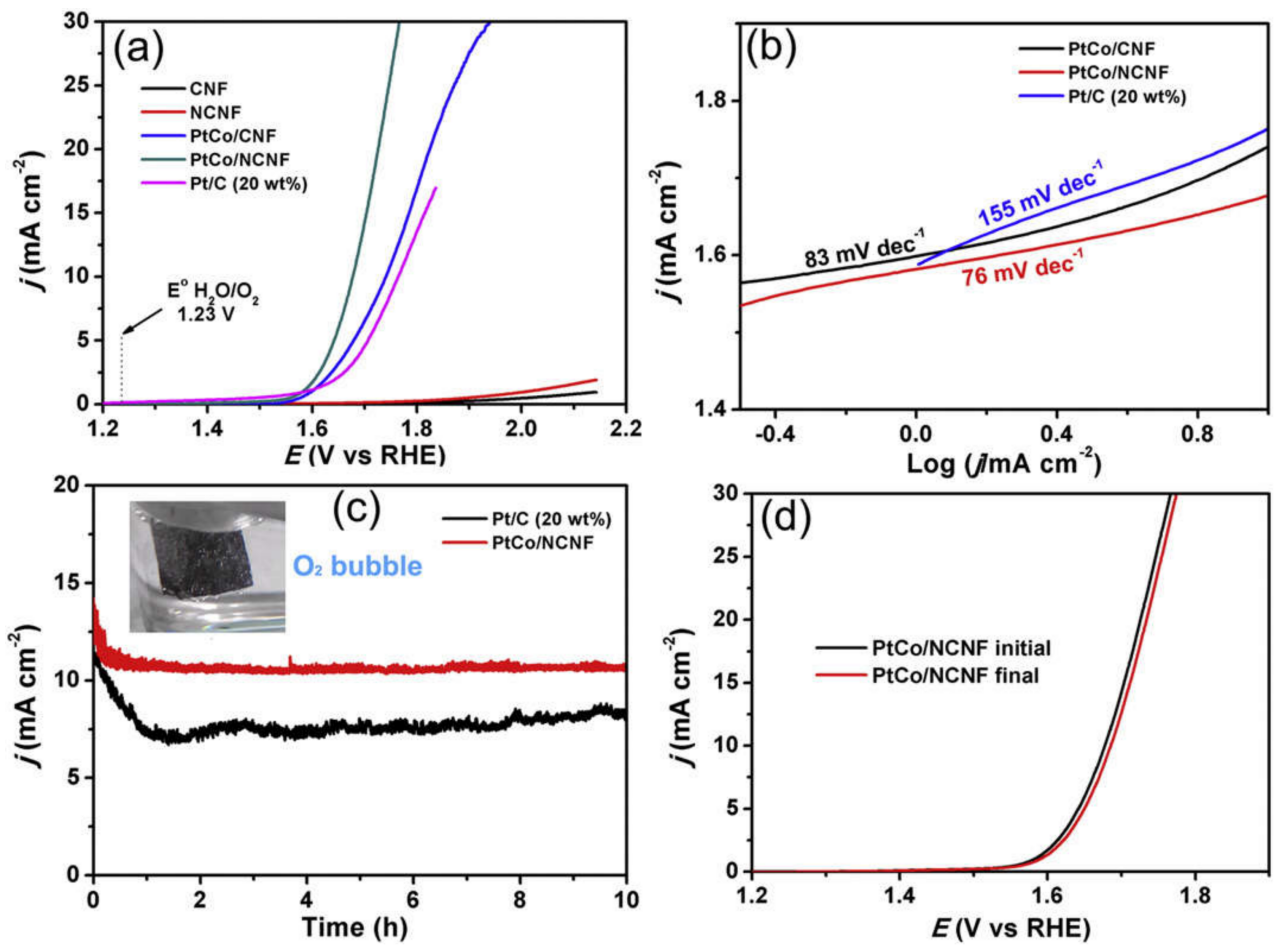
OER activity of a hybrid PtCo and N-doped carbon nanofiber catalyst in 1 M KOH. (**a**) Polarization curves of CNF, NCNF, PtCo/CNF and PtCo/NCNF, as well asPt/C catalyst (20 wt%), at a potential sweep rate of 1 mV s^−1^. (**b**) Tafel plots of PtCo/CNF and PtCo/NCNF in 1 M KOH solution. (**c**) Chronoamperometric response (j-t) recorded on the PtCo/NCNF and Pt/C catalyst (20 wt%) membrane electrode at a constant applied potential of 1.64 V vs. RHE. The PtCo/NCNF catalyst was directly used as the working electrode. Inset in (**c**) is the optical image of the PtCo/NCNF membrane electrode operating at 1.64 V with generated O_2_ bubbles on the surface. (**d**) Polarization curves of the PtCo/NCNF membrane electrode before and after 1000 potential cycles (scan rate: 100 mV s^−1^). Copyright 2016 by Elsevier [52].

Liu et al., constructed a hybrid electrocatalyst for OER application by the amalgamation of PtCo and N-doped carbon nanofiber (NCNF) [52]. The hybrid PtCo/NCNF comprises PtCo alloy nanoparticles (NPs) surrounded by a few N-doped carbon layers. The SEM images illustrate that PtCo alloy NPs exist with an average diameter of 8.9 nm, and they are uniformly embedded in the carbon matrix. The hybrid material was tested towards catalytic performance for OER activity and the results were compared with other combinations of materials as well as the well-known commercial catalyst Pt/C, as depicted in Figure 7. The PtCo/NCNF catalyst showed the OER reaction at 1.64 V vs. RHE compared to the N-undoped PtCo/CNF (1.74 V), and this indicates that the N content tremendously enhanced the activity (Figure 7a). All other materials exhibited a very poor catalytic response with anobserved potential around 1.9 V.

Further, the catalytic response of PtCo/NCNF was compared with the commercial Pt/C and found that PtCo/NCNF displayed 0.12 V lower potential than Pt/C (1.76 V). The electron transfer kinetics was investigated by the Tafel slopes and reported that 76, 83 and 155 mV dec^−1^were achieved for PtCo/NCNF, PtCo/CNF and Pt/C (20 wt%), respectively (Figure 7b). This clearly specifies that the interaction between PtCo alloy NPs and the embedded N-doped carbon layer plays a significant role in improving the electron transfer efficiency and catalytic response.The stability of PtCo/NCNF was investigated by applying a constant potential at 1.64 V in 1 M KOH for 10 h (Figure 7c). No significant difference was noted in the current density for PtCo/NCNF, whereas the commercial Pt/C catalyst dropped the current density after the continuous operation for 10 h. Further, the durability of the PtCo/NCNF was evaluated by continuous cycling between 0 and 1.2 V. As shown in Figure 7d, the current density decreased a negligible amount after 1000 cycles, and it confirms that PtCo/NCNF has long-term viability under the operating conditions. The observed superior catalytic OER activity of PtCo/NCNF is associated with the synergistic effects of the small and well-dispersed carbon core-shell structures of the PtCo alloy and the high conductivity of N-doped CNF.

Boron (B) doping with nanomaterials is also considered an imperative method for developing efficient electrode materials for OER application. The boron is electrophilic and induces a positive charge on the neighboring C atom when integrating with the carbon materials. It has been reported that the introduction of boron into graphene leads to develop of σ bonds by three valence electrons of boron with three electrons of the C atom from graphene [53]. Extra π-electrons present in the C can create a deficiency of electrons in the conduction band and produce a p-type character in the valence band. A result of decreasing electron density in the boron is that it can directly increase the electron density of the neighboring active carbon, and this promotes the catalytic activity of B-doped materials [54]. Joshi et al., reported the fabrication of electrode materials for OER activity by the integration ofIrO_2_ nanoparticles with B-doped reduced graphene oxide [55]. They demonstrated the effect of B-doping on the electronic structure modification of the IrO_2_ nanoparticles. When the doping of B was at 2 wt %, the OER catalytic current density reached 28.6 mA cm^−2^ at 1.60 V for IrO_2_-B-rGO. The observed current density was almost 8-fold higher than that of B-free IrO_2_-rGO. Additionally, the current density was 5 times higher in the B-doped electrode than pristine IrO_2_ powder.

Li et al., employed a hydrothermal-assisted biomimetic strategy to prepare the hybrid electrode material such as a CoS_2_ nanodot-embedded heteroatoms (nitrogen and sulphur)-doped carbon layer (CoS_2_@HADC) [56]. The catalyst was prepared by exposing the material at 200 °C for a different time frame. For instance, the catalysts obtained at 8, 10 and 12 h are denoted as CoS_2_@HADC-8 h, CoS_2_@HADC-10 h and CoS_2_@HADC-12 h, respectively. The catalytic performance towards OER was evaluated in 1 M KOH. All hybrid catalysts exhibited an improved catalytic response compared to the individual counterpart CoS_2_. As reported, the optimized hybrid CoS_2_@HADC-10 showed the lowest overpotential of 226 mV at 10 mA/cm^2^, compared to the CoS_2_ (256 mV vs. RHE), CoS_2_@HADC-8 h (236 mV) and CoS_2_@HADC-12 h (236 mV). Additionally, the commercial catalyst RuO_2_ achieved the overpotential of 320 mV, which is about 94 mV higher when compared to the CoS_2_@HADC-10. Further, the Tafel slope value was calculated to be 56, 93, 75 and 74 mV dec^−1^ for CoS_2_@HADC-10, CoS_2_, CoS_2_@HADC-8 h and CoS_2_@HADC-12 h, respectively.

**Figure 8:**
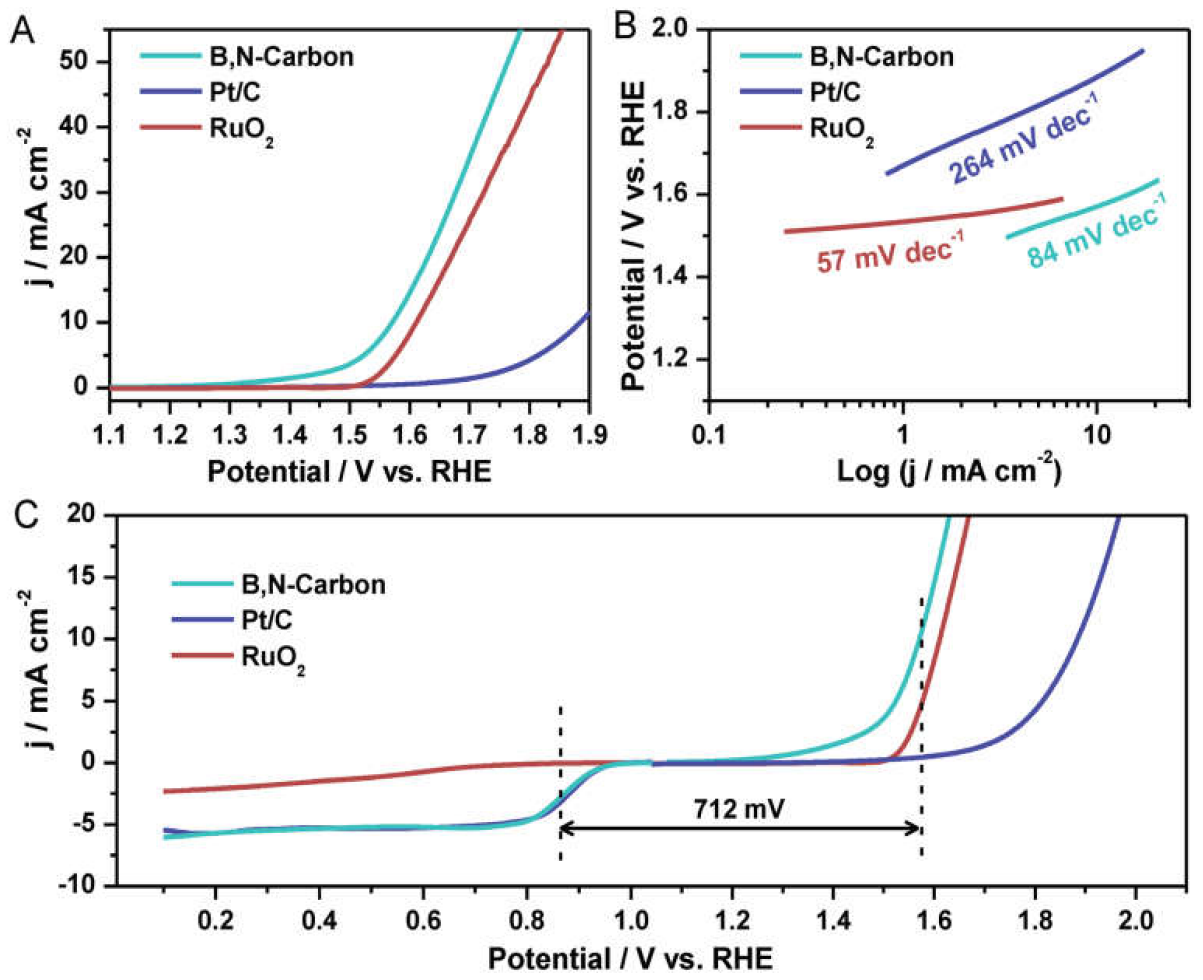
OER performances of the catalysts. (**a**) LSV curves measured at a rotation speed of 1600 rpm with a scan rate of 5 mV s^−1^. (**b**) Tafel plots. (**c**) Bifunctional catalytic activities for ORR and OER in O_2_-saturated 1.0 M KOH. Copyright 2018 by Wiley-VCH Verlag GmbH & Co. Weinheim [57].

A bifunctional catalyst was developed for the OER and ORR based on B- and N-doped nanocarbon material [57]. The material was obtained by pyrolyzing the mixture of ethyl cellulose (EC) and 4-(1-naphthyl) benzene boronic acid (NBBA) in a NH_3_ atmosphere. To achieve a higher catalytic activity, carbon defects are generated by the decomposition of EC and Zn-based templates containing oxygen species into CO_x_ and H_2_O by the graphitization process. The morphology of the nanocarbon composite was examined by the SEM, and it was found that it appeared as a nano sheet of a few hundred nanometers in size with a thickness of 20–30 nm. The fabricated material was successfully applied to the OER activity and also compared the catalytic performance with the conventional precious catalysts such as RuO_2_ and Pt/C. Figure 8, shows the onset potential of 1.57, 1.61 and 1.89 V vs. RHE obtained for B- and N-doped carbon, RuO_2_ and Pt/C, respectively. It was clear that B- and N-doped carbon material exhibited a lower overpotential for OER activity than the other two commercial catalysts. Additionally, a similar ORR performance was achieved for B- and N-doped carbon when compared to Pt/C. It was revealed that the higher catalytic response of B- and N-doped carbon is associated with the presence of high-density carbon defects, which help to activate the graphitic π-electron system [58] along with the adsorption of oxygen species.

### 4.3. Metal-Doped Electrodes

**Figure 9:**
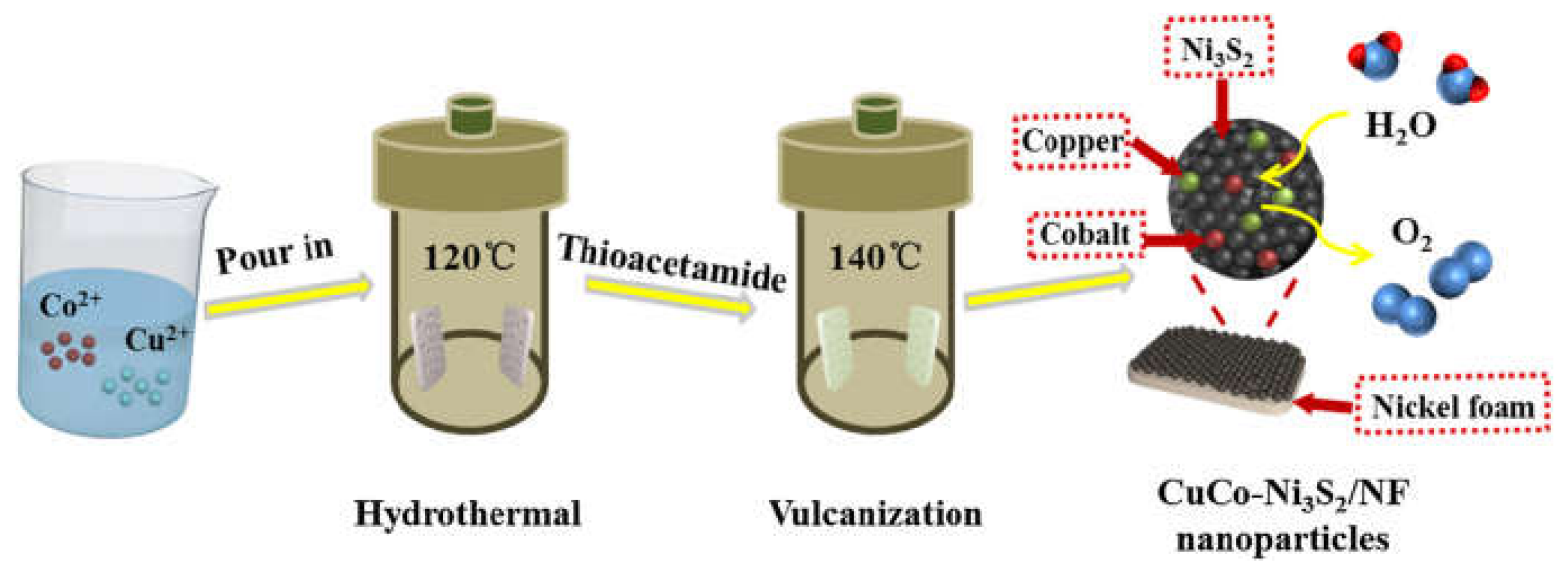
Fabrication processes of CuCo-Ni_3_S_2_/NF. Copyright 2020 by Elsevier [62].

In general, transition metals are tremendously used as electrode materials for many applications [59,60,61]. This is because they are highly conductive due to the presence of multiple oxidation states, provide excellent redox properties and the proton is easily inserted or removed into the crystal lattice through the reduction-oxidation process. A binder-free electrode material was constructed based on copper (Cu) and cobalt (Co) co-doped Ni_3_S_2_ (CuCo-Ni_3_S_2_) coated on Ni foam (NF) [62]. Simple hydrothermal and liquid-phase vulcanization methods were employed to directly deposit the Ni_3_S_2_ NPs on the NF surface, as illustrated in Figure 9. The catalytic activity of fabricated CuCo-Ni_3_S_2_/NF and its monometallic counterparts was investigated towards OER in 1 M KOH. The catalytic response was also compared with commercial catalyst RuO_2_/NF. A very poor catalytic response was observed for the bare NF electrode. On the other hand, a pronounced OER activity was recorded with CuCo-Ni_3_S_2_/NF and showed the overpotential of 400 mV vs. RHE at a current density of 100 mA cm^−2^. All other electrode materials, such as Co-Ni_3_S_2_/NF, Cu-Ni_3_S_2_/NF, Ni_3_S_2_/NF and RuO_2_/NF, exhibited the overpotentials of 425 mV, 430 mV, 438 mV and 460 mV, respectively. In addition to the OER catalytic peak, oxidation appeared at 1.4 V, which was attributed to the Ni^2+^/Ni^3+^. Further, the activity of the catalyst was optimized based on the Cu to Co ratio and it was realized that the 1:1 ratio showed superior catalytic activity for OER. The observed superior catalytic performance of CuCo-Ni_3_S_2_ is due to co-doping of Co and Cu ions. It was mentioned that Co ion doping enhances the catalytic active sites, and Cu ion doping facilitates the charge transfer rate.

A monolithic electrocatalyst was designed for OER activity based on the electrodeposition of a rare-earth praseodymium-doped nickel−cobalt−iron hydroxide (NiFeCoPrO) onto a gold-deposited nickel foam (NF) substrate [63]. NiFeCoPrOAu/NF appeared in a size range between 2 and 80 nm, with a peak volume of ~3.5 nm. Cyclic voltammetry was used to investigate the step-by-step incorporation of Co, Pr and Au. The catalytic performance towards OER activity was examined in 3 M KOH. NiFeCoPrOAu/NF showed a well-defined redox wave around 1.3 V vs. RHE, corresponding to theNi^2+^/Ni^3+^ and Co^3+^/Co^4+^ redox couples [64]. The estimated overpotentials were 228, 247, 251, 260 and 393 mV for NiFeCoPrOAu/NF, NiFeCoPrO/NF, NiFeCoO/NF, NiFeO/NF and bare NF, respectively, at a current density of 60 mA cm^−2^. Among the electrode materials, the lowest onset potential was achieved at NiFeCoPrO-Au/NF. These results supported that the incorporation of Pr and Au could remarkably increase the catalytic activities. A Tafel slope of 28 mV dec^−1^was obtained for the NiFeCoPrO-Au/NF electrode, which is significantly smaller than the commercial catalysts RuO_2_ (52.3 mV dec^−1^) and IrO_2_ (63.1 mV dec^−1^).

Transition metals, such as Co, Fe, Mn and Mo, were doped onto nickel phosphide NP-based electrode catalysts [65]. The as-prepared pristine and metal-doped nickel phosphide NP-coated GC electrodes were evaluated for OER performance in 1 M KOH. The pristine nickel phosphide NPs exhibited the overpotential of 0.39 V vs. RHE at 20 mA cm^−2^. On the other hand, the transition metal-doped catalysts achieved the overpotential of 0.33, 0.41, 0.55 and 0.43 V for FeCoP, NiCoP, NiMnP and NiMoP, respectively. Among the catalysts, FeCoP displayed a better catalytic response owing to providing an optimal bonding interaction with M–O through the filling of antibonding and bonding bands.

Morever, a NiCoFe-layered double hydroxides catalyst was developed by the co-precipitation method for bifunctional activities such as ORR and OER. The catalytic performance ofNiCoFe was evaluated by varying the Ni:Co:Fe ratios such as M1 (33%Ni:33%Co:34%Fe), M2 (27%Ni:40%Co:33%Fe) and M3 (47%Ni:21%Co:32%Fe) [66].M3 exhibited the lowest onset potential, and the highest catalytic response among these ratios compared tothe other two catalysts, and the catalytic activity followed in the order M3 < M1 < M2.These results revealed that the higher Ni content in the M3 promotes the synergy effect between the NiO amount and spinel-type structure.

## 5. Water Splitting Electrolyzer for OER

The energy essential for an indelible life is rising on a daily basis [67,68]. Hydrogen, which is well known as an environmentally benign energy carrier, has piqued the interest of researchers over the last few decades due to the fact that its oxidation product is only water [69,70,71]. Therefore, high hydrogen utilization as fuel, formed by artificial photosynthesis or electrolysis, can diminish the reliance on fossil fuels as well as the CO_2_ emission [72]. Consequently, water splitting is indeed one of the fundamental hydrogen manufacturing units for the aforesaid fuel consumption [73]. The water splitting is often based on two half-reactions: water oxidation and reduction, as shown below.

4H^+^ + 4e^−^ → 2H_2_ E = (0–0.059 pH) V vs. SHE (1)

2H_2_O → O_2_ + 4H^+^ + 4e^−^ E = (1.23 V – 0.059 pH) V vs. SHE (2)

Overall: 2H_2_O → 2H_2_ + O_2_ ∆E = –1.23 V vs. SHE (3)

**Figure 10:**
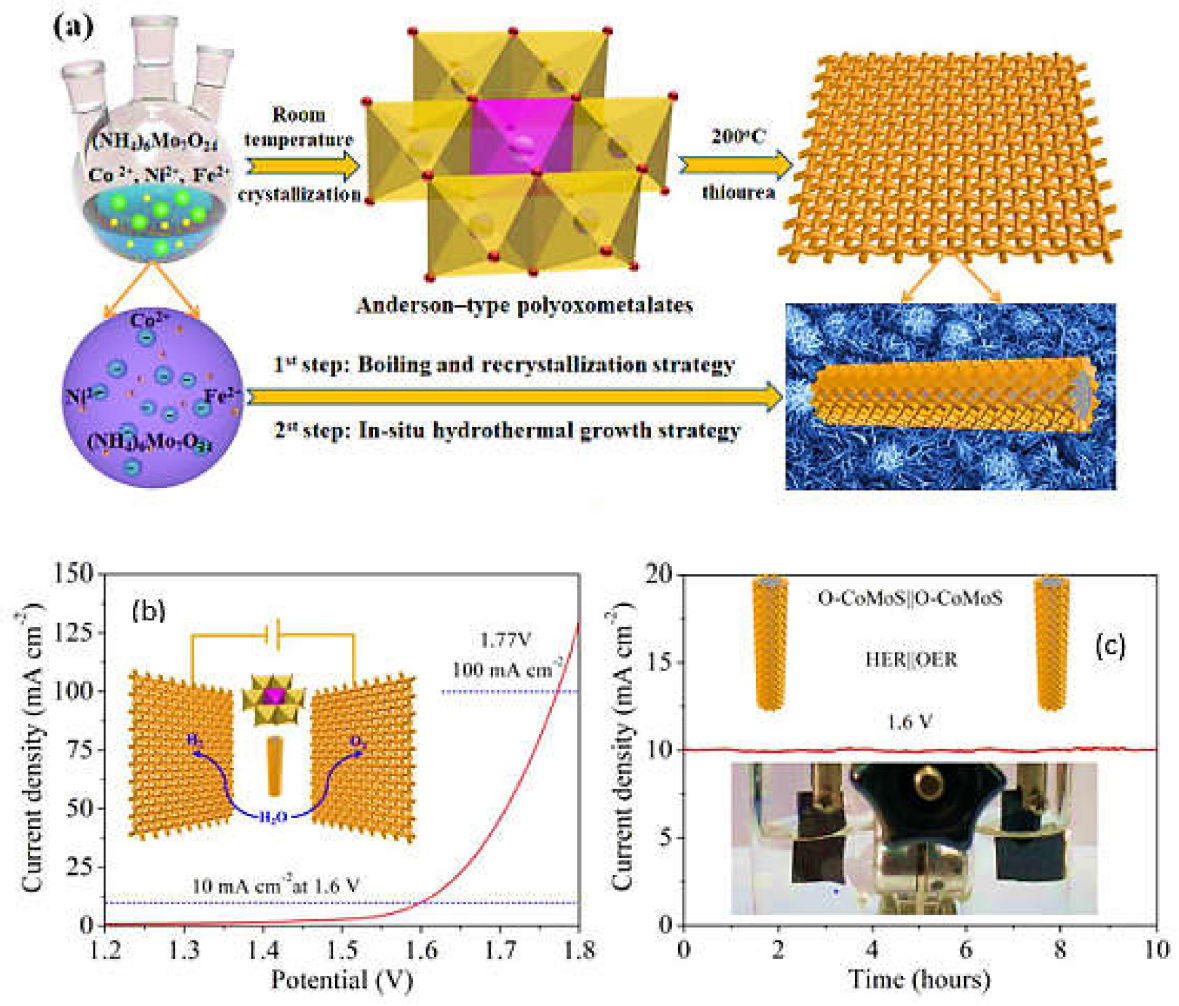
(**a**) Schematic illustration of the synthesis of as-prepared oxygenated-bimetal-sulfide hetero-nanosheet arrays grown on carbon fiber cloth by use of Anderson–type(NH_4_)_4_[M^II^Mo_6_O_24_H_6_]·6H_2_O poly oxo metalates. (**b**) Overall water splitting performance and (**c**) time dependence of current densities under different constant potentials by the use of an O-CoMoS array as a perspective anode and cathode for overall water splitting. Copyright 2018 by American Chemical Society [78].

Four protons from the water molecule are removed, resulting in the formation of O=O bonds during the water oxidation reaction, and at low potential, the activity of the water oxidation reaction is low, which is a significant hindrance for water splitting [74,75]. Numerous catalysts have been established to improve the water oxidation activity at a low potential. Due to their superior performance, noble metals are at the top of the list. Ni-based materials are promising electrocatalysts for the OER for water splitting in alkaline media. Gautam et al. synthesized various compositions of metallic and bimetallic Ni-Cu-nanostructured electrocatalysts for the OER. The Ni-Cu bimetallic nanoclusters of a specific molar ratio (52:48 mol% Ni-Cu NSc) had an OER onset overpotential of 50 mV and an overpotential of 150 mV at 10 mA cm^2^, making them the most effective nonprecious metal OER catalysts [76]. A simple solvothermal method was used to grow the NiSe/CFs catalyst. The prepared catalyst has excellent electrocatalytic activity and stability for OER in alkaline solutions. For instance, with a current density of 50 mA cm^−2^, the overpotential is 318 mV, and the Tafel slope achieved is 46 mV dec^−1^. At a current density of 20 mA cm^−2^ for 120 h, a stable catalyst is formed. The electron transfer channel offered by an ordered nanowire structure and the enhanced catalytic sites afforded by NiSe have improved the OER catalytic performance. These noble metal-based electrocatalysts in alkaline solutions might be beneficial in the development of renewable energy sources [77]. Hou et al. [78] grew vertically aligned oxygenated-CoS_2_-MoS_2_ (O-CoMoS) hetero-nanosheets on a flexible carbon fiber cloth using Anderson-type (NH_4_)_4_[Co(II)Mo_6_O_24_H_6_] 6H_2_O polyoxometalate as bimetal precursors (Figure 10a).

**Figure 11:**
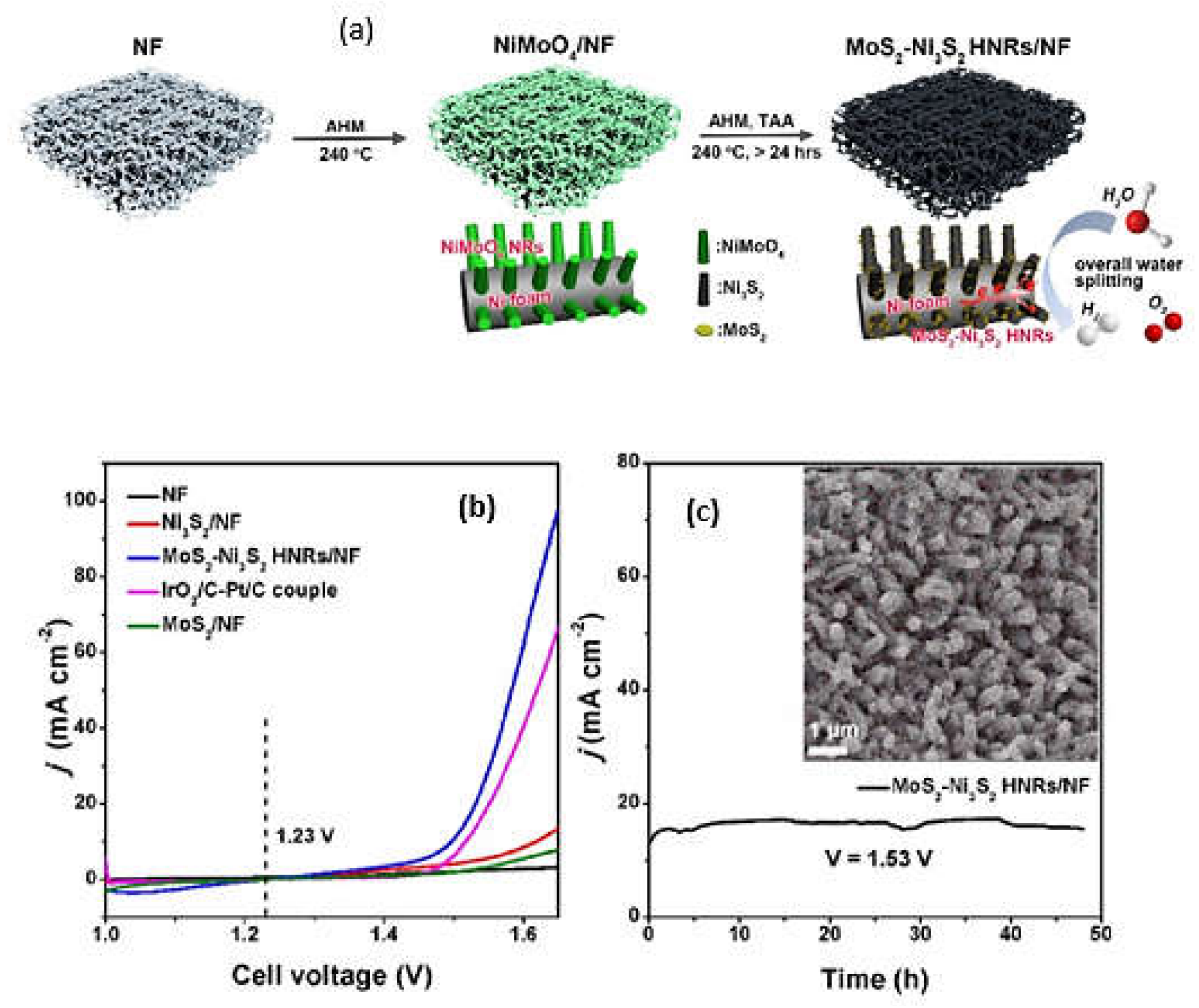
(**a**) Schematic representation of the fabrication of hetero-structured MoS_2_–Ni_3_S_2_ HNRs/NF composites. (**b**) Polarization curves and (**c**) long-term durability tests at 1.53 V of MoS_2_−Ni_3_S_2_ HNRs/NF for overall water splitting in a 1.0 M KOH solution. Inset: the SEM image of MoS_2_−Ni_3_S_2_ HNRs/NF after water electrolysis for 48 h. Copyright 2017 by American Chemical Society [79].

The improved OER performance the O-CoMoS nanosheet array had an enhanced overpotential of 272 mV to deliver a current density of 10 mA cm^−2^ (Figure 10b). O-CoMoS exhibited an outstanding OER performance among the nanosheets prepared corresponding to the overpotentials of 286, 301 and 310 mV at the current densities of 20, 50 and 100 mA cm^−2,^ respectively (Figure 10c). The highest electrocatalytic performance of O-CoMoS architecture may be attributed to structural disorder, the active heterointerfaces exposure, the active catalytic sites and charge transport between the two conductive channels. Correspondingly, MoS_2_–Ni_3_S_2_ HNRs/NF has been synthesized successfully via a simple hydrothermal method in which anisotropic molybdate intermediates are used to direct the development of hierarchical MoS_2_–Ni_3_S_2_ hetero structures (Figure 11a).

The prepared MoS_2_–Ni_3_S_2_ electrocatalysts performed better in terms of OER and HER, as demonstrated by the low overpotential values of 98 and 249 mV, respectively. The MoS_2_–Ni_3_S_2_ HNRs/NF can also be used as a bifunctional electrocatalyst for overall water splitting in a two-electrode system (Figure 11a,b). Interestingly, it provides a current density of 10 mA cm^−2^in 1.0 M KOH at a cell voltage of 1.50 V, i.e., an amalgamated overpotential of ~270 mV. Furthermore, when the applied voltage was adjusted to 1.53 V, the electrolyzer provided a smooth line with a consistent current density of ~17 mA cm^−2^ for 48 h (Figure 11c). It also persisted after the overall water splitting for 48 h, showing the acceptable enduring durability of MoS_2_–Ni_3_S_2_ HNRs under the strong alkaline electrolyte [79]. A few striking findings on OER catalytic activity under acidic conditions were made. The authors analyzed the IrNi(Ox) catalyst forms in the literature and identified that IrNi HT had the second-highest mass activity [80]. Shan et al., inferred a while back that the co-doped RuIr alloy considerably improved OER activity and stability under acidic conditions due to optimal oxygen-intermediate binding behavior and a lower reduced metal dissolution compared to the RuIr alloy [81]. In another study, You et al. [82] developed 3D-hierarchically porous urchin-like Ni_2_P microsphere superstructures anchored on nickel foam as bifunctional electrocatalysts for overall water splitting. The Ni_2_P/Ni/NF catalysts were prepared by a phosphidation process. The optimal Ni_2_P/Ni/NF demonstrated exceptional catalytic performance and stability for OER in alkaline electrolytes. When used as an OER electrocatalyst, Ni_2_P/Ni/NF was largely oxidized to nickel oxides/hydroxides/oxyhydroxides on the catalyst surface and showed outstanding OER activity with low overpotentials of 200 and 268 mV reaching 10 and 100 mA cm^−2^, respectively. Evans et al. [83] devised a new electrochemical technique for synthesizing stoichiometric, phase pure CoSb_2_O_6_ and MnSb_2_O_6_ electrodes to examine their capability to oxidize water in acidic media. When the same geometric area was employed, the electrode CoSb_2_O_6_ performed better; however, the performance of MnSb_2_O_6_ was superior when the electrochemically active surface was considered. Furthermore, both demonstrated consistent V-t performance during the OER at 10 mA/cm^2^ after a considerable time of 24 h of V-t measurement. Du et al. [84] successfully prepared a polyhedron transition metal-organic framework carbon nanomaterial (Co/P/MOFs-CNTs-700) co-doped with phosphorus and carbon sources that can be used as an efficient and cheap bi-functional electrochemical catalyst. The Co/P/MOFs-CNT-s-700 exhibited the lowest overpotential of 420 mV, which achieved the current density of 10 mA cm^−2^ for OER, and the half potential was 0.8 V for ORR in 0.1 M NaOH, which is extremely similar to commercial electrochemical catalysts. It might be used as a potential electrochemical bifunctional electrocatalyst in the energy storage industry, as well as providing a promising insight into electrochemical bifunctional electrocatalyst design.

## 6. Energy Conversion for OER

One of the most important concerns nowadays is energy. As conventional fossil fuels become depleted, so many socio-ecological issues have arisen. A practical solution to this challenge is the development of green and renewable energy. Previously, sophisticated electrochemical energy conversion devices, like as fuel cells [85] and water splitting [86] were extensively researched in order to produce a cost-effective, supportable, consistent, and environmentally friendly substitute to traditional energy systems. Enhancing the performance of electrochemical energy conversion systems, including ORR [87], OER [88], HER, [89] and other technologies, has taken a lot of research and engineering work. Many developed electrode materials could catalyze two or more reactions simultaneously; however, most work focuses on improving the electrocatalytic activity of a single reaction, while ignoring the synthesis of a multifunctional electrocatalyst with high activity and good longevity [90]. Herein, we discussed some of the electrocatalysts for energy conversion systems that involve OER. In alkaline conditions, Ni and its alloys are perhaps the most effective catalysts for the OER [91,92], whereas Ir and Ru are the most efficient catalysts in acidic media [93,94].

Bizzotto et al., presented a novel, reliable and scalable approach for the synthesis and evaluation of extremely tiny and monodisperse Ir NPs with outstanding OER performance as catalysts. This catalyst is a potential option for the development of supported catalysts with an exceptionally high electrochemically active surface area due to the small Ir NPs size and the stability of the colloidal suspension [95]. ORR and OER are the two critical processes involved in metal-air batteries. Particularly, in Zn-air batteries, these processes can create a significant impact on the total efficiency of the charge and discharge cycle. Theelectrocatalyst NC-Co SA, comprising Co single atoms anchored in porous N-doped carbon flakes, has been synthesized by a carbonization acidification method. Co-MOF was employed as a precursor and the resulting material is very sustainable.

**Figure 12:**
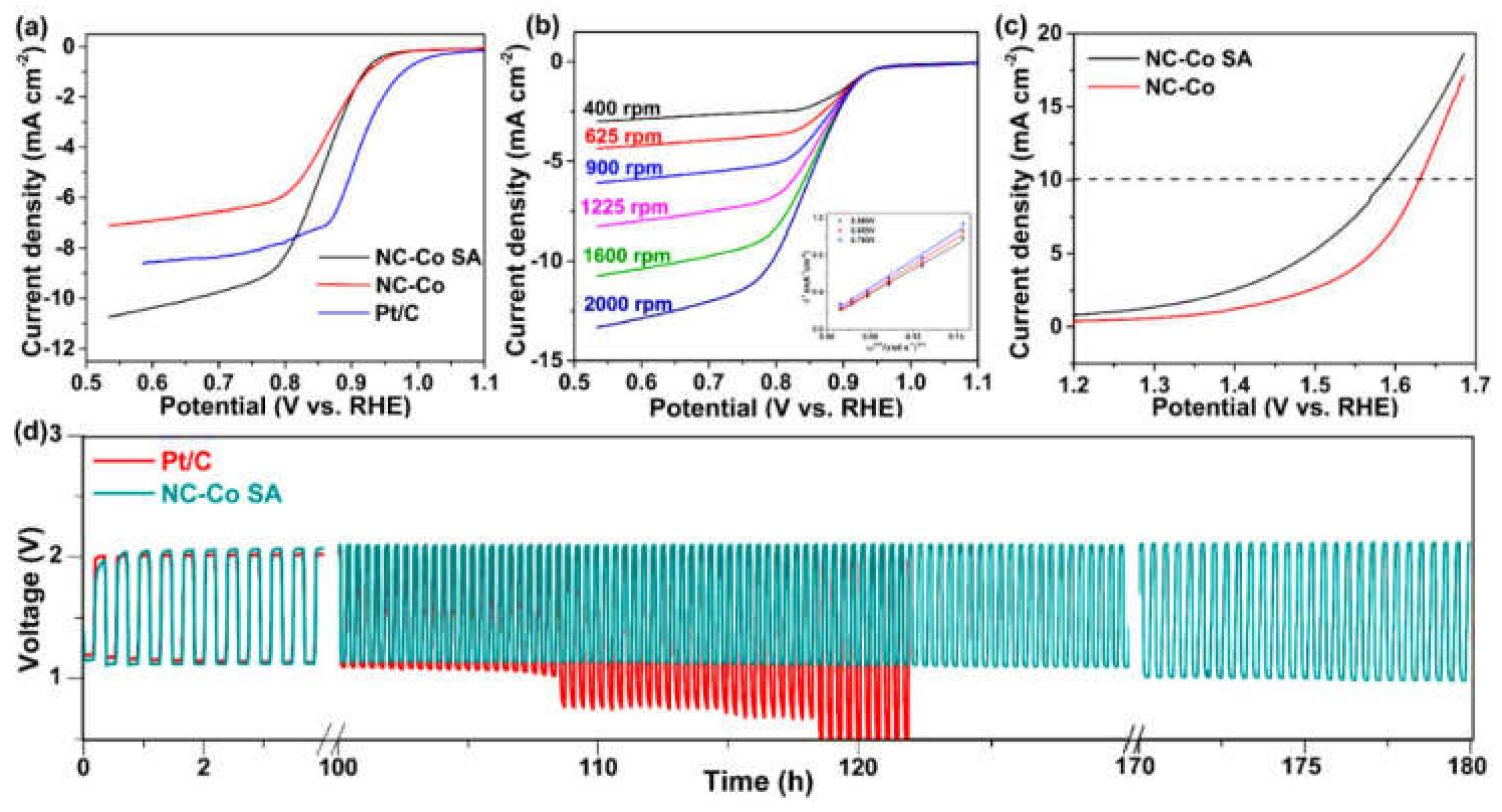
(**a**) Oxygen reduction polarization curves at the rotation speed of 1600 rpm. (**b**) Oxygen reduction polarization curves of NC-Co SA at different rotation speeds and Koutecky–Levich plots. (**c**) Oxygen evolution curves. (**d**) Stability test of aqueous rechargeable Zn-air batteries using NC-Co SA and Pt/C as the air cathodes. Copyright 2018 by American Chemical Society [96].

As depicted in Figure 12a, although NC-Co SA’s onset potential (E_onset_ = 1.00 V, E_1/2_ = 0.87 V, vs. RHE) and half-wave potential are similar to those of NC-Co (E_onset_ = 1.02 V, E_1/2_ = 0.86 V, vs. RHE), its saturation current at 0.60 V (vs. RHE) may reach 10.38 mA cm^−2^, which is significantly higher than that of NC-Co (6.93 mA cm^−2^) and the precious Pt/C catalyst (8.52 mA cm^−2^). Under three diverse voltages, the K-L plots reveal a better linear relationship between i^−1^ and ω^−0.5^ (Figure 12b). In terms of the OER catalytic activities displayed in Figure 12c, when the current density approaches 10 mA cm^−2^, NC-Co SA exhibits a lower overpotential (360 mV) than thePt/C catalyst (401 mV), demonstrating its potential OER activities. The battery using NC-Co SA as the cathode showed no voltage change (Figure 12d) after 570 charge/discharge cycles (equal to 180 h), which was more stable than the battery with Pt/C after 325 cycles (equal to 108 h), demonstrating the former’s outstanding cycling stability.

The single-atom catalysts have plenty of potential for non-noble metal-based electrocatalysts for flexible energy conversion and storage devices [96]. LaNiO_3_ and LaNi_0.85_Mg_0.15_O_3_ nanofibers were synthesized via an electrospinning method. The prepared LNMO NFs had more OER and ORR activity than LaNiO_3_, as well as a higher positive half-wave potential (0.63 V) and a lower overpotential of 0.45 V at a current density of 10 mA cm^−2^. Because the LNMO has a strong oxygen-binding surface that enhances OER activity, it could be employed as a promising bifunctional catalyst for zinc-air battery applications [97]. Exploration of non-noble metals and high-activity electrocatalysts via simple and controlled techniques continues to be a significant problem for zinc-air batteries. Cheng et al., adopted a melt polymerization strategy to synthesize iron polyphthalocyanine metallic-organic frameworks over the carbon black matrix. Through non-covalent π–π interactions, FePPc molecules may be anchored on the carbon black matrix, facilitating the electron transfer process and system stability. Because of the adequate free electrons and MN_4_ catalytic sites in the macroscale structure, the as-synthesized FePPC@CB displays enhanced OER electrocatalytic activity. Furthermore, it also performs well in liquid and flexible all-solid-state batteries [98].

**Figure 13:**
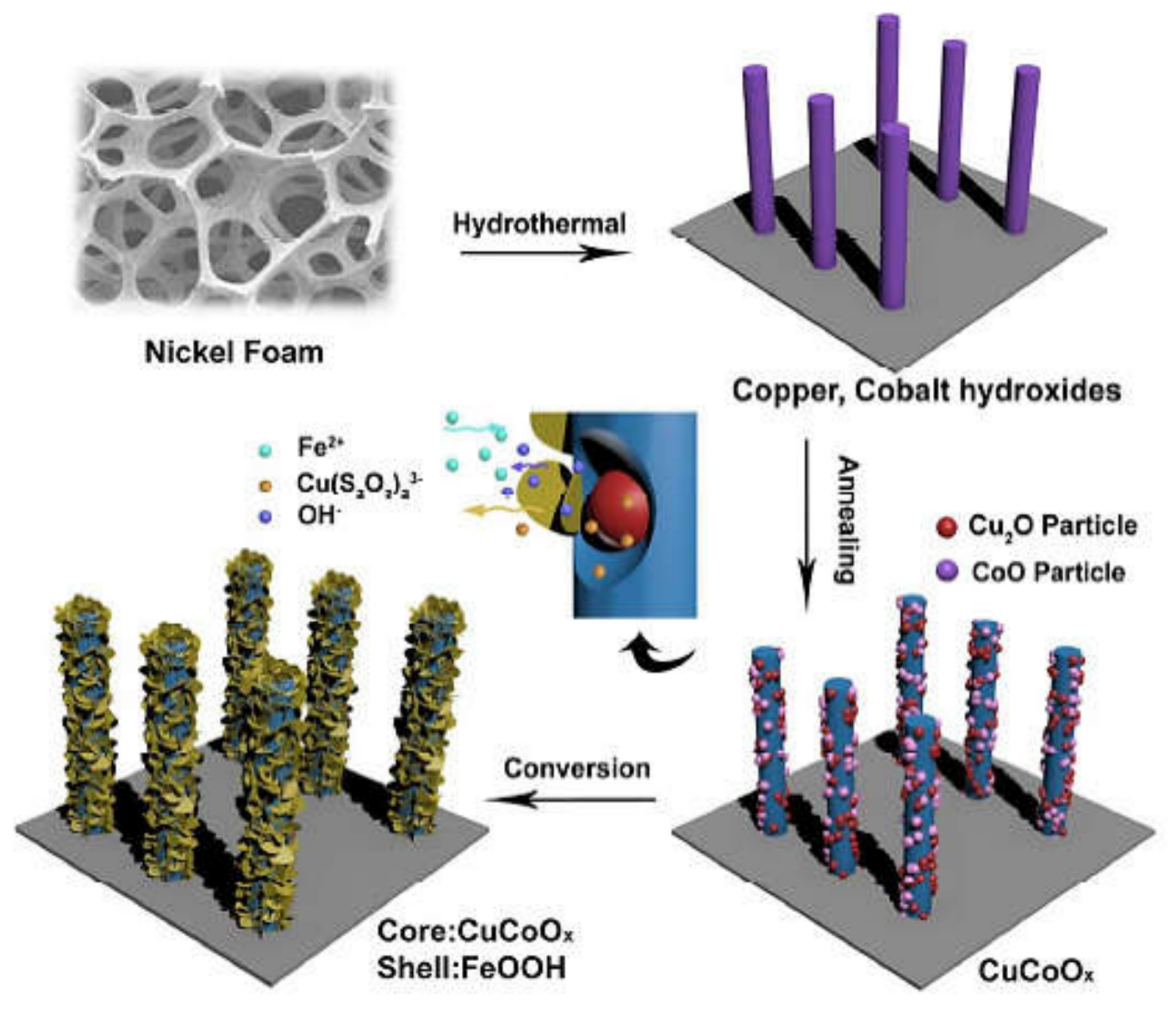
Schematic illustration showing the fabrication of the CuCoO_x_/FeOOH hybrid nanowire electrocatalysts, including the growth, annealing and conversion synthesis process. Copyright 2017 by American Chemical Society [101].

In recent decades, fuel cells have made tremendous progress in energy applications [99]. For fuel cells, it is essential to have an oxygen electrocatalyst that is both efficient and stable. Metal nitride nanosheets were prepared by Tian et al. [100] using a simple hydrothermal method. The Ti_0.8_Co_0.2_N nanosheets exhibited substantial oxygen reduction activities in both acidic H_2_-air and alkaline Zn-air fuel cells. Hence, the nanosheets may be employed as a potential application in the real alkaline metal-air fuel cells. Interestingly, Kuang et al. [101] developed copper, cobalt-based oxide/iron hydroxide hybrid nanowire arrays (CuCoO_x_/FeOOH) through a three-step growth/annealing/conversion process (Figure 13).

A rechargeable Zn-air battery with a moderate charge/discharge overpotential (0.75 V at 10 mA cm^−2^) and long-term cycling stability (150 cycles) was constructed using the CuCoO_x_/FeOOH electrocatalyst as the oxygen electrode, indicating novel bifunctional electrocatalysts for energy conversion and storage applications. Hierarchical porous double-shelled (MgCo)_3_O_4_ electrocatalysts with controllable Mg substitution have been synthesized via an insitu method. Mg substitution, in particular, improves electrical conductivity and affects electrocatalysis by inducing lattice buffer zones. The findings of this study provide an encouraging insight into the rational design of efficient oxygen electrocatalysis for real-world energy conversion systems [102].

## 7. Conclusions and Future Perspectives

In this article, we focused on the development of new types of efficient electrocatalysts for OER applications, owing to the easy formation, surface engineering, cost-effective and regulating energy conversion and energy storage system. In summary, we highlighted the most dynamic natured metal oxides embedded onto functionalized carbon and conductivepolymer-based materials, which can help realize their catalytic activity and long-term stability towards OER. More precisely, we systematically analyzed OER electrocatalysts with massive electrocatalytic activity, desirable mass activity, rapid energy conversion and storage technologies that are still challenging. With this perspective, the focused nanocomposite’s structure activity relationship will get a new approach and better understanding for the development of sustainable energy resources. Consequently, we discussed advanced electrocatalysts that could provide new promising energy sources for OER, as well as their general mechanisms and outcomes in terms of catalytic performance, low overpotential, electrode stability and Tafel slope measurment. The simple and cost-effective routes for the fabrication of functionalized nanocomposite materials should be developed and the detailed mechanistic aspects are highlighted herein. More emphasis on the various nanostructured electrocatalysts have significantly proved their promise in fuel cell catalysts. The applications of highly conductive natured CNF, Co_3_O_4_, 3D graphene and NiCo_2_O_4_ in OER have led to improvements in their catalytic activity. In particularly, the construction of highly monodispersed and structurally ordered electrocatalysts will have major research impacts in the future.

## Data Availability

Data are contained within the article or the Supplementary Materials.

## Supplementary Materials

The following are available online at https://www.mdpi.com/article/10.3390/ma14164420/s1, Table S1: Summary of OER electrocatalytic activities of the summarized different kinds of advanced electrocatalysts.
