# Supplementary material for: Recent Progress in the Development of Advanced Functionalized Electrodes for Oxygen Evolution Reaction: An Overview
Source: Materials (Basel). 2021 Aug 6;14(16):4420. doi: 10.3390/ma14164420 (PMC8400293; doi:10.3390/ma14164420)
Supplement: Supplementary file 1 [file materials-14-04420-s001.zip › materials-1299801-supplementary.pdf]

Review

Recent Progress in the Development of Advanced Functionalized Electrodes for Oxygen Evolution Reaction: An Overview

Tse-Wei Chen [1], Palraj Kalimuthu [2], Ganesan Anushya [3], Shen-Ming Chen [4,*], Vinitha Mariyappan [4] and Rasu Ramachandran [5,*]

1 Department of Materials, Imperial College London, London SW7 2AZ, UK; t.chen19@imperial.ac.uk
2 School of Chemistry and Molecular Biosciences, University of Queensland, Brisbane 4072, Australia; p.kalimuthu@uq.edu.au;
3 Department of Physics, S.A.V. Sahaya Thai Arts and Science (Women) College, Sahayam Nagar, Kumarapuram Road, Vadakkankulam, Tirunelveli-627116, India; anushya@savsahayathaicollege.com
4 Electroanalysis and Bioelectrochemistry Lab, Department of Chemical Engineering and Biotechnology, National Taipei, University of Technology, No.1, Section 3, Chung-Hsiao East Road, Taipei 106, Taiwan; vinithavicky80@gmail.com
5 Department of Chemistry, The Madura College, Vidya Nagar, Madurai-625011, India
* Correspondence: smchen@ntut.edu.tw (S.-M.C.); ramachandran@maduracollege.edu.in (R.R.)

Table S1. Summary of OER electrocatalytic activities of the reported various types of advanced electrodecatalysts.

Synthesis route	Catalysts	Overpotential (mV)	Tafel slope (mV dec^{-1})	Current density (mA cm^{-2})	Medium (M)	Ref.
Electrochemical	(P-PbO_2)-MnO_2	~139	196	50	1.63 H_2SO_4	[1]
Electrochemical	Mesoporous Ni film	463	126	10	6 KOH	[2]
Pyrolysis	Ir-based catalyst	243	92	1.40	0.1 $HClO_4$	[3]
Self-assembled	2D-NiO/CeO_2	212	60	10	1 KOH	[4]
Hydrothermal	Trimetallic NiFeCr	225	69	25	1 KOH	[5]
Hydrothermal	Ni@$NiCo_2O_4$	270	67	10	1 KOH	[6]
Self-assembled	N-Co_3O_4	1180	29.8	10	1 KOH	[7]
Hydrothermal	Mesoporous $NiCo_2O_4$	~350	43	10	1 KOH	[8]
Hydrothermal	AuNP/$CoMoN_x$	166	46	10	1 KOH	[9]
Hydrothermal	Carbon-enriched	207	97	10	1 KOH	[10]

References

1. Li, Y.; Jiang, L.; Liu, F.; Li, J.; Liu, Y.; Novel phosphorous doped PbO_2-MnO_2bicontinuous electrodes for oxygen evolution reaction. RSC Adv.**2014**, 4, 24020-24028.

2. Farmani, A.L.; Nasirpouri, F.; Boosting hydrogen and oxygen evolution reactions on electrodeposited nickel electrodes via simultaneous mesoporosity, magnetohydrodynamics and high gradient magnetic force. J. Mater. Chem. A **2020**, 8, 24782-24799.

3. Ko, J.S.; Johnson, J.K.; Johnson, P.I.; Xia, Z.; Developing oxygen and chlorine evolution reaction in sea water using iridium-based electrocatalysts. Chem. Cat. Chem. **2020**, 12, 4526-4532.

4. Zhang, Y.; Ye, F.; Li, W.; Self-assembled two-dimensional NiO/CeO_2 heterostructure rich in oxygen vacancies as efficient bifunctionalelectrocatalyst for alkaline hydrogen evolution and oxygen evolution. Chem. Eur. J. **2020**, 27, 3766-3771.

5. Yang, Y.; Dang, L.; Shearer, M.J.; Sheng, H.; Li, W.; Chen, J.; Xiao, P.; Zhang, Y.; Hamers, R.J.; Jin, S.; Highly active trimetallic NiFeCr layered double hydroxide electrocatalysts for oxygen evolution reaction. Adv. Energy. Mater. **2018**, 1703189.

6. Wang, L.; Gu, C.; Ge, X.; Zhang, J.; Zhu, H.; Tu, J.; Decorating $NiCo_2O_4$ shell on a hollow Ni nanorod array core for water splitting with enhanced electrocatalytic performance. Chem. Nano. Mater. **2017**, doi: 10.1002/cnma.201700291.

7. Li, X.; Wei, J.; Li, Q.; Zheng, S.; Xu, Y.; Du, P.; Chen, C.; Zhao, J.; Xue, H.; Xu, Q.; Pang, H.; Nitrogen-doped cobalt oxide nanostructures derived from cobalt-alanine complexes for high performance oxygen evolution reactions. Adv. Funct. Mater. **2018**,

1800886.

8. Broicher, C.; Zeng, F.; Artz, J.; Hartmann, H.; Besmehn, A.; Palkovits, S.; Palkovits, R.; Facile synthesis of mesoporous nickel cobalt oxide for OER-insights into intrinsic electrocatalytic activity. Chem. Cat. Chem. **2018**, doi: 10.1002/cctc.201801316.

9. Yao, R.Q.; Shi, H.; Wan, W.B.; Wen, Z.; Lang, X.Y.; Jiang, Q.; Flexible Co-Mo-N/Au electrodes with a hierarchical nanoporous architecture as high efficient electrocatalysts for oxygen evolution reaction, Adv. Mater. **2020**, 1907214.

10. Surendran, S.; Shanmugapriya, S.; Lee, Y.S.; Sim, U.; Kalaiselvan, R.; Carbon-enriched cobalt phosphide with assorted nanostructure as a multifunctional electrode for energy conversion and storage devices, Chemistry Select. **2018**, 3, 12303-12313.
